# In Vitro Analysis of Biological Activity of Circulating Cell-Free DNA Isolated from Blood Plasma of Schizophrenic Patients and Healthy Controls—Part 2: Adaptive Response

**DOI:** 10.3390/genes13122283

**Published:** 2022-12-04

**Authors:** Svetlana V. Kostyuk, Elizaveta S. Ershova, Andrey V. Martynov, Andrey V. Artyushin, Lev N. Porokhovnik, Elena M. Malinovskaya, Elizaveta M. Jestkova, Natalia V. Zakharova, George P. Kostyuk, Vera L. Izhevskaia, Sergey I. Kutsev, Natalia N. Veiko

**Affiliations:** 1 Federal State Budgetary Scientific Institution, Research Centre for Medical Genetics, 115522 Moscow, Russia; 2 N. A. Alekseev Clinical Psychiatric Hospital No 1, Moscow Healthcare Department, 117152 Moscow, Russia

**Keywords:** schizophrenia, cell-free DNA, NOX4, NRF2, SOD1, HIF1A, γH2AX, BRCA1, adaptive response

## Abstract

Oxidized in vitro genomic DNA (gDNA) is known to launch an adaptive response in human cell cultures. The cfDNA extracted from the plasma of schizophrenic patients (sz-cfDNA) and healthy controls (hc-cfDNA) contains increased amounts of 8-oxodG, a DNA-oxidation marker. The aim of the research was answering a question: can the human cfDNA isolated from blood plasma stimulate the adaptive response in human cells? In vitro responses of ten human skin fibroblasts (HSFs) and four peripheral blood mononuclear cell (PBMC) lines after 1–24 h of incubation with sz-cfDNA, gDNA and hc-cfDNA containing different amounts of 8-oxodG were examined. Expressions of RNA of eight genes (*NOX4, NFE2L2, SOD1, HIF1A, BRCA1, BRCA2, BAX* and *BCL2*), six proteins (NOX4, NRF2, SOD1, HIF1A, γH2AX and BRCA1) and DNA-oxidation marker 8-oxodG were analyzed by RT-qPCR and flow cytometry (when analyzing the data, a subpopulation of lymphocytes (PBL) was identified). Adding hc-cfDNA or sz-cfDNA to HSFs or PBMC media in equal amounts (50 ng/mL, 1–3 h) stimulated transient synthesis of free radicals (ROS), which correlated with an increase in the expressions of *NOX4* and *SOD1* genes and with an increase in the levels of the markers of DNA damage γH2AX and 8-oxodG. ROS and DNA damage induced an antioxidant response (expression of *NFE2L2* and *HIF1A*), DNA damage response (*BRCA1* and *BRCA2* gene expression) and anti-apoptotic response (changes in *BAX* and *BCL2* genes expression). Heterogeneity of cells of the same HSFs or PBL population was found with respect to the type of response to (sz,hc)-cfDNA. Most cells responded to oxidative stress with an increase in the amount of NRF2 and BRCA1 proteins along with a moderate increase in the amount of NOX4 protein and a low amount of 8-oxodG oxidation marker. However, upon the exposure to (sz,hc)-cfDNA, the size of the subpopulation with apoptosis signs (high DNA damage degree, high NOX4 and low NRF2 and BRCA1 levels) also increased. No significant difference between the responses to sz-cfDNA and hc-cfDNA was observed. Sz-cfDNA and hc-cfDNA showed similarly high bioactivity towards fibroblasts and lymphocytes. Conclusion: In cultured human cells, hc-cfDNA and sz-cfDNA equally stimulated an adaptive response aimed at launching the antioxidant, repair, and anti-apoptotic processes. The mediator of the development of the adaptive response are ROS produced by, among others, NOX4 and SOD1 enzymes.

## 1. Introduction

Cell-free DNA (cfDNA) is a term for DNA fragments circulating in the extracellular internal medium of the human body. In recent decades, cfDNA circulating in human peripheral blood has attracted growing attention from researchers. Blood cfDNA fragments are isolated from blood serum or plasma. The source of cfDNA in the blood are the body’s cells that have died earlier due to necrosis or apoptosis. In addition, the living cells excrete DNA fragments into the medium in the form of particles of various size [1,2,3,4,5,6,7].

Circulating cfDNA is widely used in diagnostics. In the total pool of cfDNA, DNA originated from a tumor, fetus or transplanted organ is assayed. The total cfDNA content and the concentration of its specific fragments are markers of the body’s status. The cfDNA content substantially increases in case of pregnancy pathology, tumor, critical state, affective or physical stress, and during intensive physical loads [8,9,10,11,12,13,14,15]. The cfDNA is also elevated in mental disorders, in particular in schizophrenia patients [16,17,18,19].

CfDNA is not a biologically neutral agent. Due to the altered GC-ratio and the levels of methylation and oxidation compared to intracellular DNA, cfDNA acquires advanced capacities to govern the functional activity of the body’s cells [20,21,22,23,24,25,26,27,28]. In our previous study on cultured skin fibroblasts in vitro, we showed that cfDNA isolated from human blood plasma stimulated the known DNA-sensors, which control the signaling pathways modulating the immune response [29].

The present study is Part 2 of that research to continue the in vitro investigation of biological activity of cfDNA obtained from plasma of a schizophrenia patient (sz-cfDNA) and healthy controls (hc-cfDNA). In contrast to the previous study [29], we explored the response in two cell types: cultured human skin fibroblasts (HSFs) and peripheral blood lymphocytes (PBL). Since the cfDNA samples isolated from blood plasma had been shown to contain oxidized DNA fragments [17,29], we supposed that hc-cfDNA and sz-cfDNA could induce the adaptive response in cultured cells.

It was found earlier that model samples of oxidized DNA (oxy-gDNA), in contrast to non-oxidized gDNA, induced an adaptive response in human cells, which is similar to the cells’ response to small doses of ionization radiation [30,31,32,33,34,35,36,37]. The response includes the transient induction of reactive oxygen species (ROS) synthesis by NOX4 (NADPH-oxidase 4) pro-oxidant enzyme. NOX4 is the cellular oxidoreductase that catalyzes hydrogen peroxide generation. It is the major factor of oxidative stress in a number of disorders [38]. Significant expression of NOX4 gene was found in the lymphocytes obtained from SZ patients [39].

NOX4 is known to contribute to the antioxidant response via modulating *NFE2L2* gene expression and affecting the activity of nuclear factor erythroid 2-related factor 2 (NRF2) in cells influenced by endo- and exogenous deleterious factors. The NRF2 transcription factor is the master regulator of the antioxidant enzyme expression [40,41].

*NFE2L2* gene was demonstrated to be tightly connected with the *SOD1* gene [42]. Superoxide dismutase-1 (SOD1), which metabolizes superoxide radicals to hydrogen peroxide and molecular oxygen, is a major cytoplasmic antioxidant enzyme [43].

*NFE2L2* gene activation was previously shown to be an initiating signal for *HIF1A* activation. An important role in maintaining cellular oxygen homeostasis is played by hypoxia-inducible transcription factor 1-α (HIF-1A). The reciprocal impact of HIF-1A and NRF2 is coordinated during hypoxia or oxidative stress [44,45,46].

The transient oxidation of cellular DNA and the formation of double-strand breaks was also previously shown to result from an ROS burst induced by model oxy-gDNA. The main product of DNA oxidation is 8-oxo-2′-deoxyguanosine (8-oxodG), an important biomarker of oxidative DNA damage in vivo. The 8-oxodG content reflects the oxidative stress intensity [47,48]. Phosphorylated on serine 139, histone H2AX (γH2AX) is a marker of double-strand DNA breaks in human studies at population level [49].

Cellular DNA damage is known to activate the genes of double-strand DNA break repair. *BRCA1* and *BRCA2* genes are normally expressed in the cells. They help either repair damaged DNA or kill the cell when DNA cannot be repaired. Involved in the repair of chromosomal damage, these genes play a key role in the error-free repair of double-strand DNA breaks [50,51].

In case of non-repaired damage, the cell launches the program of apoptosis. As shown earlier, model fragments of oxy-gDNA induced the antiapoptotic response in the cells. B-cell lymphoma 2 (BCL2) protein regulates cell death via inhibiting apoptosis [52]. The major apoptotic activator is BCL2 Associated X (BAX) protein, which has been shown to participate actively in p53-mediated apoptosis [53]. The Bcl2/Bax expression ratio appears to determine the output of an apoptotic stimulus: death or survival [54].

Thus, the investigation of a hypothetic adaptive response that HSFs and PBL develop to the actions of hc-cfDNA and sz-cfDNA fragments included the examination of pro- and anti-oxidative responses (changes in the *NOX4, NFE2L2, SOD1* and *HIF1A* gene expression that is involved in ROS metabolism), antiapoptotic response (expression of *BAX* and *BCL2* genes) and DNA damage response (DNA damage markers 8-oxodG and γH2AX, and expression of DNA repair genes *BRCA1* and *BRCA2*). As a result of our study, we obtained one more confirmation of the hypothesis of the role of circulating cfDNA in the peripheral blood as an important bystander factor of the cell’s adaptive response to internal and external adverse impacts.

## 2. Results

### 2.1. DNA Fragments

The fraction of the DNA oxidation marker 8-oxodG as part of DNA samples varied from 0.5 8-oxodG/10^6^ N (gDNA) up to 24 (hc-cfDNA) and 65 (sz-cfDNA). The length of DNA fragments varied from 6 up to 10 kb. The cfDNA samples contained 1–2% shorter DNA fragments (200 b) [29].

### 2.2. Exposure to (hc,sz)-cfDNA Induced an Increase in ROS Production

**Figure 1:**
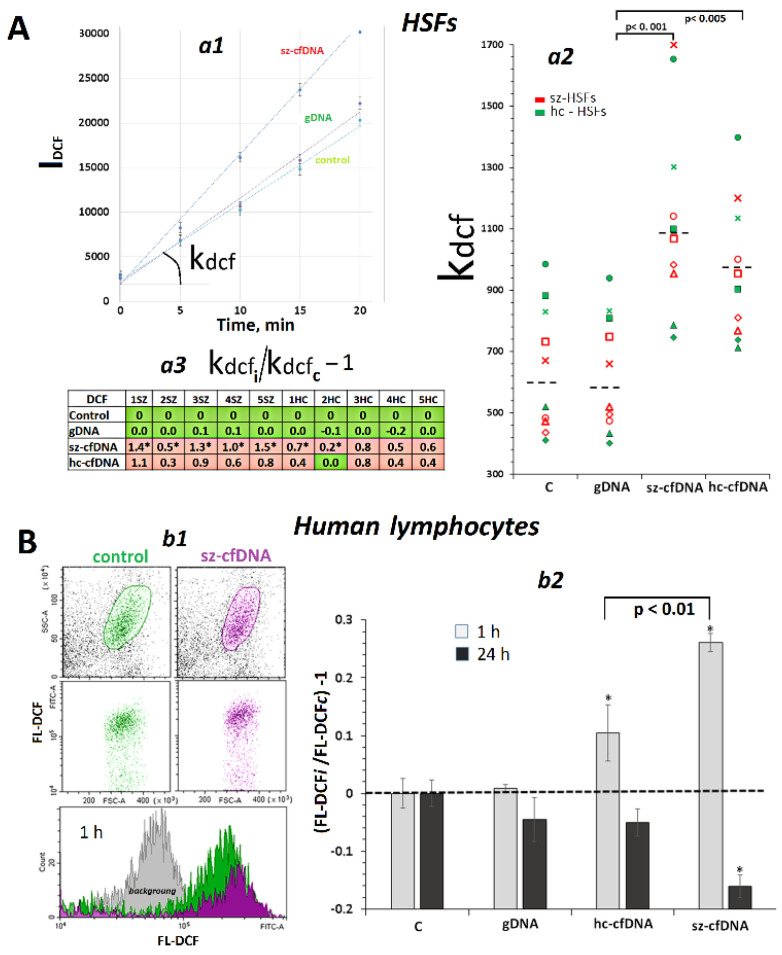
The exposure to cfDNA leads to an increase in ROS production. (**A**). Synthesis of ROS in HSFs, which were incubated for 1 h in the presence of DNA samples (50 ng/mL). *a1*—The results of the quantification of fluorescence using plate reader. The time kinetics of fluorescence outputs in cells treated with H_2_DCFH-DA. The data from the device are given for sz-HSF3. *a2*—Change of the k_dcf_ in the presence of DNA samples. The DNA samples are indicated on the graph; medians—dotted lines. *a3*—Analysis of relative changes in ROS levels in the presence of DNA samples compared to control. Green color—no significant differences with the control (*p* > 0.01), red—increased RNA content in the presence of the DNA sample (*p* < 0.01). (*) the response to sz-cfDNA differs from the response to hc-cfDNA for this HSF (*p* < 0.01). (**B**). Synthesis of ROS in PBL, which were incubated for 1 and 24 h in the presence of DNA samples. *b1*—The most typical examples of ROS assay with FCA in PBL. *b2*—Change of the ROS level in PBL in the presence of DNA samples (50 ng/mL). Average values for four lymphocyte samples and the standard deviation are given. (*)—the differences with the control are significant (*p* < 0.01).

Changes in ROS production in HSFs exposed to the three DNA samples were assayed using H2DCFH-DA reagent and a tablet fluorescent reader (Figure 1). The kinetics of DCF fluorescence increase were analyzed after adding the reagent to cells which had been being incubated for 1 h with DNA samples (Figure 1A*a1*). Figure 1A*a2* presents k_dcf_ constants that indicated the ROS content in the cells. Changes in the constants after the exposure to DNA samples compared to the reference samples are shown in Figure 1A*a3.*

No significant difference was found in the HSF response to exposure to the three DNA samples between sz-HSFs and hc-HSFs cell lines (*p* > 0.1). The hc-cfDNA and sz-cfDNA samples induced a substantial increase in ROS production by a factor of 1.1–2.5 compared to the controls and to gDNA (Figure 1A*a2*). In 7 of 10 HSFs, sz-cfDNA induced a stronger response than hc-cfDNA (Figure 1A*a3*).

Changes in ROS content in lymphocytes (n = 4) after exposure to DNA samples were analyzed using FCA with H2DCFH-DA reagent (Figure 1B). In 1 h, hc-cfDNA and sz-cfDNA samples stimulated the elevation of ROS content in PBL compared to controls and the gDNA sample (Figure 1B*b2*). In 24 h, we observed a decrease in cellular ROS content as compared to controls. The sz-cfDNA-induced ROS changes, both burst in 1 h and subsequent decrease in 24 h, were stronger than those induced by hc-cfDNA samples.

Thus, hc-cfDNA and sz-cfDNA samples, in contrast to gDNA samples, induced an early response in HSFs and PBL, which included a jump in ROS production. In order to understand the cause of the changes of ROS production upon the cfDNA exposure, we monitored the activity of some genes which have an effect on cellular ROS metabolism.

### 2.3. NOX4 Expression

#### 2.3.1. In *HSFs*

**Figure 2:**
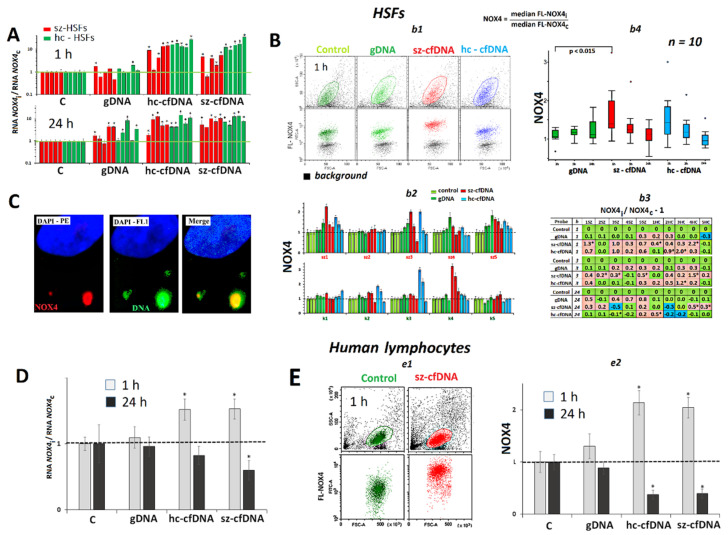
The exposure of HSFs and PBL to cfDNA (50 ng/mL) leads to the changes in *NOX4* expression. (**A**). Change of the RNA *NOX4* in HSFs in the presence of DNA samples. Average values for three measurements and standard deviation are given. The cultivation time and the DNA samples are indicated on the graph. The response to cfDNA differs from the control HSFs (*p* < 0.01). (**B**). *b1*—The most typical examples of NOX4 assay with FCA in HSFs. The data from the device are given for sz-HSF3 (1 h). *b2*—Index NOX4: the values of the medians of FL-NOX4i, normalized to the control signal value. Average values for three measurements and standard deviation are given. *b3*—Analysis of relative changes in NOX4 levels in the presence of DNA samples compared to control. Figures indicate the ratio (NOX4 DNA sample—NOX4control)/NOX4control. Green color—no significant differences with the control (*p* > 0.01), red—increased RNA content in the presence of the DNA probe (*p* < 0.01) and blue—decreased RNA content (*p* < 0.01). (*) the response to sz-cfDNA differs from the response to hc-cfDNA for this HSF (*p* < 0.01). *b4*—Changes in the NOX4 index in sample of HSFs (n = 10). (**C**). Localization of the labeled DNA sample (green) and NOX4 (red) in HSFs. An example is given for the sz-HSF3 line (probe sz-cfDNA, 1 h). (**D**). Change of the RNA NOX4 in PBL in the presence of DNA samples (50 ng/mL). Average values for four lymphocyte samples and standard deviation are given. (**E**). *e1*—The most typical examples of the NOX4 analysis with FCA in lymphocytes. *e2*—Changes in the NOX4 index in sample of lymphocytes (n = 4).

An hour after adding hc-cfDNA and sz-cfDNA to the HSF medium, we observed a multifold increase of *NOX4* RNA in 9 of 10 HSFs as compared to the controls (Figure 2A). There were no differences between the reactions for hc-cfDNA and sz-cfDNA (*p* > 0.1). Twenty-four hours after adding sz-cfDNA and hc-cfDNA samples, the elevated *NOX4* RNA content, which exceeded the reference level in each cell line, was retained in HSFs. The gDNA sample also launched increased *NOX4* expression in 7 of 10 HSFs in 1 h or in 24 h; however, the effect was much lower (*p* < 0.0015) than in the case of hc-cfDNA or sz-cfDNA.

The NOX4 protein contents in HSFs were measured using FCA (see Figure 1B*b1*). Upon a 1-h long exposure to sz-cfDNA and hc-cfDNA samples, the mean NOX4 protein content increased in 8 of 10 HSFs compared to the controls by a factor of 1.3 to 3.2. The effect was at maximum 1 h after adding DNA samples to the medium, and decreased after 3 h of cultivation (Figure 1B*b2*–*b4*). Exposure to gDNA also induced the increase of NOX4 in 6 of 10 HSFs in 3 h; however, the effect was much lower (*p* < 0.002) than in case of hc-cfDNA or sz-cfDNA (Figure 2B*b3*). Median values of the signals (parameter m(FL-NOX4)), indicating the average content of the protein after 1 h of cultivation, positively correlated with the amount of *NOX4* RNA (Rs = +0.48, *p* = 0.0017, n = 40), Table S1. Normalized index NOX4 (m(FL-NOX4)i/m(FL-NOX4)c), which displays how many times the average cellular content of the protein changes after exposure to DNA samples compared to the reference samples, also positively correlated with the amount of *NOX4* RNA (Rs = +0.50, *p* = 0.0010, n = 40).

In 24 h of HSF incubation with hc-cfDNA and sz-cfDNA samples, NOX4 protein content decreased almost down to the reference level (Figure 2B*b3*,*b4*). In three HSFs, significant reduction of NOX4 content was observed, as compared to the reference samples. The parameters m(FL-NOX4) and index NOX4 in 24 h did not correlate with the amount of *NOX4* RNA (*p* > 0.1).

In order to determine the joint localization of cfDNA and NOX4 within the cell, sz-cfDNA samples labelled with fluorescein (Figure 2C) were used. Part of the signals from NOX4 molecules (antibodies to NOX4 (label PE), red spots) coincided with signals from cfDNA (green spots). Thus, cfDNA boosts the NOX4 protein content in the place of the contact with the cell.

#### 2.3.2. In *PBL*

The early response demonstrated by lymphocytes to the action of hc-cfDNA and sz-cfDNA samples did not differ qualitatively from the response by HSFs. In 1 h after adding a sample, the contents of *NOX4* RNA (Figure 2D) and NOX4 protein (Figure 2E) became elevated. In 24 h, *NOX4* gene expression in the lymphocytes decreased at the levels of both protein and RNA.

### 2.4. NFE2L2 Expression

***HSFs***. An hour after adding hc-cfDNA and sz-cfDNA samples to the HSF medium, we observed a multifold increase in *NFE2L2* RNA contents in all the HSFs (Figure 2A). Twenty-four hours after adding hc-cfDNA and sz-cfDNA samples, a high level of *NFE2L2* RNA, that exceeded the control level, was retained in HSFs. Exposure to gDNA also induced NOX4 expression in 7 of 10 HSFs in 1 h or in 24 h; however, the effect was much lower (*p* <0.001), than in case of hc-cfDNA or sz-cfDNA.

**Figure 3:**
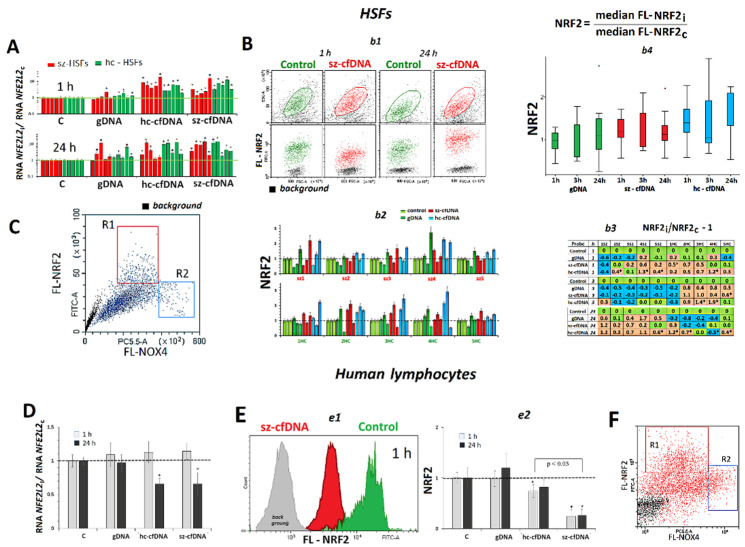
The influence of the DNA samples (50 ng/mL) on the expression of *NFE2L2* gene in HSFs and lymphocytes. (**A**). Change of the RNA *NFE2L2* in HSFs in the presence of DNA samples (50 ng/mL). Average values for three measurements and standard deviation are given. The cultivation time and the DNA samples are indicated on the graph. (*) The response to cfDNA differs from the control HSFs (*p* < 0.01). (**B**). *b1*—The most typical examples of the NRF2 assay with FCA in HSFs. The data from the device are given for sz-HSF1. *b2*—Index NRF2: the values of the medians of FL-NRF2i, normalized to the control signal value. Average values for three measurements and standard deviation are given. *b3*—Analysis of relative changes in NRF2 levels in the presence of DNA samples compared to control. Green color—no significant differences with the control (*p* > 0.01), red—increased RNA content in the presence of the DNA probe (*p* < 0.01) and blue—decreased RNA content (*p* < 0.01). (**C**). FCA. Sz-HSF4 staining with two types of antibodies with different labels: NRF2 (FITC) and NOX4 (PC5.5). (**D**). Change of the RNA *NFE2L2* in the PBL in the presence of DNA samples (50 ng/mL). Average values for four lymphocyte samples and standard deviation are given. (**E**). *e1*—The most typical examples of NRF2 assay with FCA in lymphocytes. *e2*—Changes in the NRF2 index in sample of lymphocytes (n = 4). (**F**) The amounts of NOX4 and NRF2 proteins in PBL.

The content of NRF2 protein was determined using FCA (Figure 3B). Unlike *NOX4*, the content of *NFE2L2* RNA in the cell does not correlate with m(FL-NRF2) or NRF2 index (*p* > 0.1), Table S1. HSFs responded to exposure to cfDNA samples in different ways. An hour after adding hc-cfDNA and sz-cfDNA samples to HSF culture, a decrease of NRF2 content in sz-HSF1 cell line and an increase of NRF2 were observed in five HSFs. In 3 h, the amount of the protein after exposure to cfDNA samples decreased in six HSFs and increased in four. In 24 h, the amount of NRF2 protein increased again in half of the HSF lines and decreased in the other half.

***PBL.*** The hc-cfDNA and sz-cfDNA samples induced a reduction of *NFE2L2* RNA content in 24 h of cultivation (Figure 3D) and a reduction of NRF2 protein content in 1 h and 24 h (Figure 3E). Sz-cfDNA induced a stronger fall of NRF2 than hc-cfDNA.

The cellular content of *NFE2L2* RNA in HSFs in 1 h (and 24 h) of cultivation positively correlated with the amount of *NOX4* RNA (Rs = +0.80 (+0.78), *p* < 10^−8^, n = 40), Table S1. The amount of NRF2 protein (m(FL-NRF2) index) also positively correlated with NOX4 protein content in 1 h (Rs = +0.48, *p* = 0.002, n = 40) and in 24 h (Rs = +0.64, *p* < 10^−4^, n = 40). In order to determine if the positive correlation between the expression of a pro-oxidant (*NOX4*) and anti-oxidant (*NFE2L2*) genes resulted from a simultaneous augmentation of the expression of both genes in the same cells, we measured the amounts of NOX4 and NRF2 proteins in the same HSFs (Figure 3C) and PBL (Figure 3F), using antibodies labelled with FITC (NRF2) and CY5-5 (NOX4). The analysis of plot distribution in the curve FL-NOX4—FL-NRF2 has shown that the pool of HSFs can be tentatively divided in two subsets. In most cells, an increase of NOX4 protein up to the middle level is followed by an increase in NRF2 protein from the minimum baseline up to the peak values (R1 area). However, there is a fraction of cells in the pool, which have a very high NOX4 content and a relatively low NRF2 protein content (R2 area). This fraction of HSF and PBL cell pools increased after the exposure to hc-cfDNA and sz-cfDNA samples from 1 to 4% up to 7 to 10%.

### 2.5. SOD1 Expression

**Figure 4:**
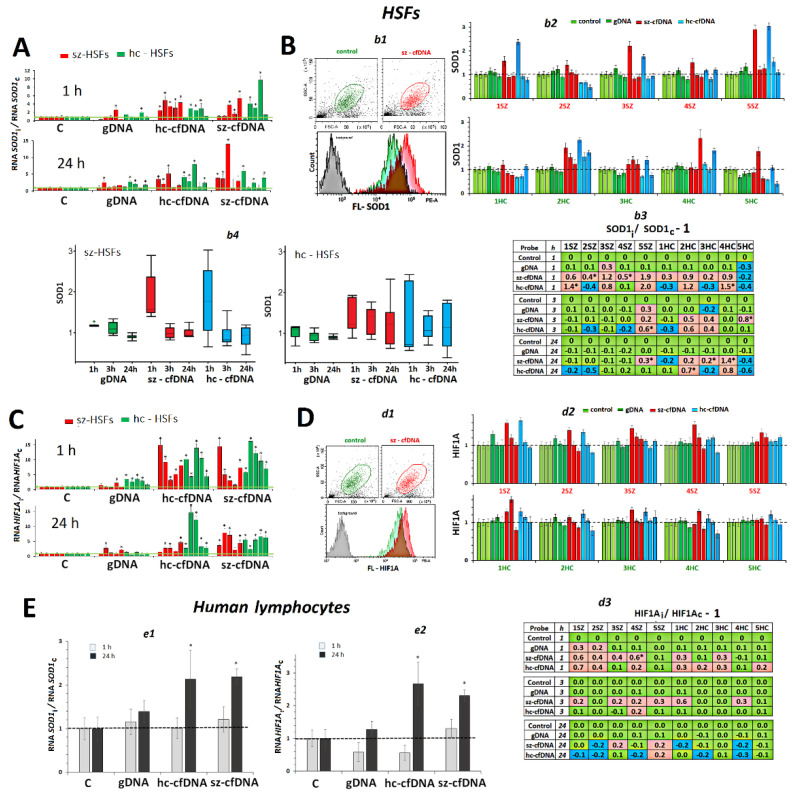
The influence of the DNA samples (50 ng/mL) on the expression of *SOD1* and *HIF1A* genes in HSFs and PBL. (**A**). Change of the RNA *SOD1* in HSFs. Average values for three measurements and standard deviation are given. The cultivation time and the DNA samples are indicated on the graph. The response to cfDNA differs from the control HSFs (*p* < 0.01). (**B**). *b1*—The most typical examples of the SOD1 assay with FCA in HSFs. The data from the device are given for sz-HSF3. *b2*—Index SOD1: the values of the medians of FL-NRF2i, normalized to the control signal value. Average values for three measurements and standard deviation are given. *b3*—Analysis of relative changes in SOD1 levels in the presence of DNA samples compared to control. Green color—no significant differences with the control (*p* > 0.01), red—increased RNA content in the presence of the DNA probe (*p* < 0.01) and blue—decreased RNA content (*p* < 0.01). *b4*—Changes in the SOD1 index in sample of sz-HSFs (n = 5) and hc-HSF(n = 5). (**C**). Change of the RNA *HIF1A* in HSFs. Average values for three measurements and standard deviation are given. The cultivation time and the DNA samples are indicated on the graph. (*) The response to cfDNA differs from the control HSFs (*p* < 0.01). (**D**). *d1*—The most typical examples of the HIF1A assay with FCA in HSFs. The data from the device are given for sz-HSF3. *d2*—Index HIF1A: the values of the medians of FL-NRF2i, normalized to the control signal value. Average values for three measurements and standard deviation are given. *d3*—Analysis of relative changes in HIF1A levels in the presence of DNA samples compared to control. Green color—no significant differences with the control (*p* > 0.01), red—increased RNA content in the presence of the DNA probe (*p* < 0.01) and blue—decreased RNA content (*p* < 0.01). (**E**). *e1* and *e2*—Change of the RNA *SOD1* and RNA *HIV1A* in the PBL in the presence of DNA samples (50 ng/mL). Average values for four lymphocyte samples and standard deviation are given.

***HSFs***. An hour after adding hc-cfDNA and sz-cfDNA samples to the HSF growth medium, we observed an increase in the *SOD1* RNA amount in the HSFs (Figure 4A). The increase did not differ statistically (*p* > 0.1) in case of hc-cfDNA and sz-cfDNA. Twenty-four hours after adding hc-cfDNA and sz-cfDNA samples, a high level of *SOD1*RNA, which exceeded the reference one, was retained in 7 HSFs. The gDNA sample also stimulated *SOD1* expression in some HSFs in 1 h or 24 h; however, the effect was much lower (*p* <0.001) than in the case of hc-cfDNA or sz-cfDNA (Figure 4A).

The amount of SOD1 protein was measured using FCA (Figure 4B). The cellular level of SOD1 protein in 1 h and 24 h did not correlate with the amount of *SOD1* RNA (*p* > 0.1), Table S1. An hour after adding hc-cfDNA and sz-cfDNA samples to the HSF culture medium, we observed an increase of the cellular SOD1 content. In 3 and 24 h, the amount of SOD1 protein decreased upon exposure to cfDNA samples (Figure 4B*b4*).

Our findings corroborate the fact of a simultaneous activation of *NFE2L2* and *SOD1* gene transcription under oxidative stress [42]. The contents of RNAs for both genes in 1 h of cultivation were correlated (Rs = +0.80, *p* <10^−8^, n = 40), as well as the contents of NRF2 and SOD1 proteins (Rs = +0.50, *p* = 0.001, n = 40).

***PBL*.** The lymphocyte response to the action of DNA samples differed from the response of HSFs by a delayed increase in the SOD1 gene expression (Figure 4E*e1*). The hc-cfDNA and sz-cfDNA samples stimulated an increase of SOD1 RNA level in 24 h of cultivation only. The gDNA sample had practically no effect on SOD1 expression.

### 2.6. HIF1A Expression

***HSFs***. In 1 h or 24 h after adding hc-cfDNA and sz-cfDNA samples to the HSF culture medium, an increase in *HIF1A* RNA amounts was observed in all the HSFs tested (Figure 4C). The increase did not differ statistically (*p* > 0.1) for hc-cfDNA and sz-cfDNA. The gDNA sample also stimulated NOX4 expression in 7 of 10 HSFs in 1 h or 24 h; however, the effect was much lower (*p* <0.001) than in case of hc-cfDNA or sz-cfDNA (Figure 4C*c2*).

The content of HIF1A protein was measured using flow cytometry (Figure 4D). The cellular HIF1A content did not correlate with an amount of *HIF1A* RNA (*p* > 0.1), Table S1. An hour after adding hc-cfDNA and sz-cfDNA samples to the culture medium, we observed an increase in HIF1A content in 6 of 10 HSFs (Figure 4D*d2*,*d3*). Index m(FL-HIF1A), which displays the average intracellular level of protein, negatively correlated with HIF1A index, which indicates the change of the protein content in comparison to the reference samples (Rs = −0.42, *p* =0.008, n = 40). In 3 and 24 h, the amount of HIF1A protein decreased under the action of the cfDNA samples. The gDNA sample had practically no effect on the expression of the *HIF1A* gene in 8 of 10 HSFs.

Our findings corroborate the fact of simultaneous activation of *NFE2L2* and *HIF1A* gene transcription under oxidative stress [44]. The levels of the RNAs for the two genes in 1 h correlated well to each other (Rs = +0.78, *p* <10^−7^, n = 40). The levels of the proteins HIF1A and NRF2 correlated in 24 h of cell incubation with DNA samples (Rs = +0.51, *p* <10^−3^, n = 40).

***PBL.*** The response of lymphocytes to the action of DNA samples differed from the response of HSFs by a delayed increase in *HIF1A* gene expression (Figure 4E*e2*). Hc-cfDNA and sz-cfDNA stimulated an increase of *HIF1A* RNA amounts in 24 h of cultivation only. The gDNA sample had practically no effect on *HIF1A* gene expression.

### 2.7. Changes in 8-OxodG Content

The elevated ROS content in the cells (Figure 1), together with a considerable activation of transcription of genes that modulate ROS content (Figure 2, Figure 3 and Figure 4), suggests that damage to cellular DNA may occur upon exposure to cfDNA.

**Figure 5:**
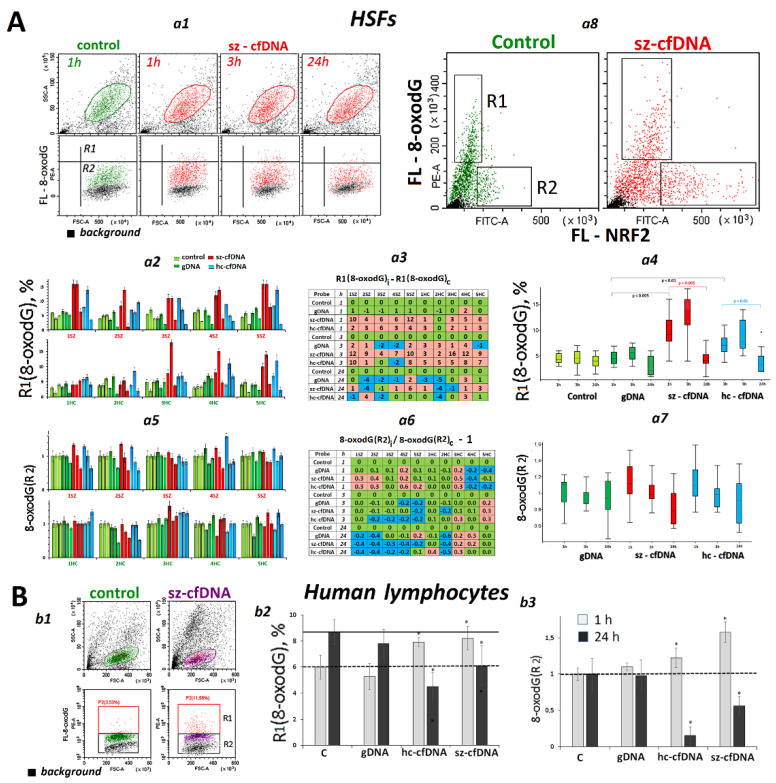
The influence of the DNA samples on the 8-oxodG levels in HSFs and PBL. (**A**). *a1*—The most typical examples of the 8-oxodG assay with FCA in HSFs. The data from the device are given for sz-HSF2. *a2*—Proportion of R1 fraction cells. *a3*—Analysis of the changes in R1(8-oxodG) levels in the presence of DNA samples compared to control. *a4*—Changes in the R1(8-oxodG) levels in sample of HSFs (n = 10). *a5*—Index 8-oxodG(R2): the values of the medians of 8-oxodG(R2), normalized to the control signal value. Average values for three measurements and standard deviation are given. *a6*—Analysis of relative changes in 8-oxodG(R2) levels in the presence of DNA samples compared to control. *a7*—Changes in the 8-oxodG(R2) levels in sample of HSFs (n = 10). *a8*—Dependence of R1(8-oxodG) on 8-oxodG(R2). (**B**). *b1*—The most typical examples of the 8-oxodG assay with FCA in lymphocytes. *b2*,*b3*—Changes in the R1(8-oxodG) index and 8-oxodG(R2) index in sample of lymphocytes (n = 4). The asterisk * marks significant differences (*p* < 0.01 when n = 10 and *p* < 0.05 when n = 4).

***HSFs***. The oxidation marker 8-oxodG was quantified using FCA (Figure 5A). The HSF cell pool contained two cell types: those with relatively high (R1) and low (R2) 8-oxodG content (Figure 5A*a1*). Two parameters were determined—a fraction of R1 cells (Figure 5A*a2*–*a4*) and median values of FL- 8-oxodG (R2) normalized by the corresponding reference (8-oxodG index, Figure 5A*a5*–*a7*). In 1 or 3 h after adding hc-cfDNA and sz-cfDNA samples to the culture medium, the fraction of R1 (8-oxodG,%) increased in each HSF. Augmentation of 8-oxodG index (R2) was found in six HSF lines only. After a prolonged incubation of HSFs with DNA samples added, the 8-oxodG content decreased down to the reference baseline and lower, showing a cyclic nature of oxidant-antioxidant balancing counteraction [55].

In order to determine if the amount of NRF2 protein has an effect on the DNA oxidation rate in HSFs, we quantified 8-oxodG, the oxidation marker, and NRF2 protein in the same HSF cells (Figure 5A*a8*) using antibodies labelled with FITC (NRF2) and PE (8-oxodG). The analysis of plot distribution in the FL-NRF2—FL-8-oxodG curve has shown that the pool of HSFs can be tentatively divided in two subsets. In most cells, an increase of NRF2 protein up to the middle level was followed by an increase of 8-oxodG from minimum to maximum values (R1 area). However, there is a fraction of cells in the pool which have a very high NRF2 content and a relatively low 8-oxodG content (R2 area). This fraction of such cells in HSF culture elevated upon exposure to hc-cfDNA and sz-cfDNA samples from 5 to 10% up to 15 to 20%.

***PBL***. Hc-cfDNA and sz-cfDNA, but not gDNA samples, stimulated an increase of DNA oxidation level in 1 h of cultivation. In 24 h, the oxidation marker content decreased below the reference level (Figure 5B).

### 2.8. Changes in γH2AX Content

In order to estimate the nuclear DNA damage degree, we assayed the amount of γH2AX protein [49].

**Figure 6:**
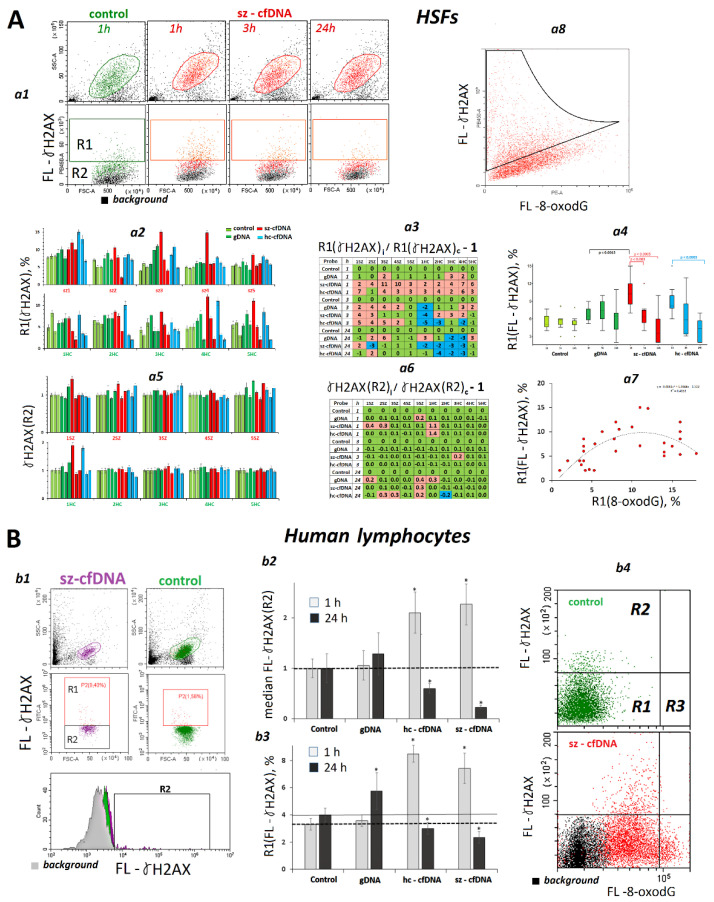
The influence of the DNA samples on the γH2AX levels in HSFs and PBL. (**A**). *a1*—The most typical examples of the γH2AX assay with FCA in HSFs. The data from the device are given for sz-HSF3. *a2*—Proportion of R1(γH2AX) fraction cells. *a3*—Analysis of relative changes in R1(γH2AX) levels in the presence of DNA samples compared to control. *a4*—Changes in the R1(γH2AX) levels in sample of HSFs (n = 10). *a5*—Index γH2AX (R2): the values of the medians of γH2AX (R2), normalized to the control signal value. Average values for three measurements and standard deviation are given. *a6*—Analysis of relative changes in γH2AX (R2) levels in the presence of DNA samples compared to control. *a7*—Dependence of R1(8-oxodG) on R1(γH2AX). *a8*—sz-HSF4 staining with two types of antibodies with different labels: γH2AX (PB450) and 8-oxodG (PE). (**B**). *b1*—The most typical examples of the γH2AX assay with FCA in lymphocytes. *b2*,*b3*—Changes in the R1(γH2AX) index and γH2AX (R2) index in sample of lymphocytes (n = 4). *b4*—PBL staining with two types of antibodies with different labels: γH2AX (PB450) and 8-oxodG (PE).

***HSFs.*** The γH2AX was quantified using FCA (Figure 6A). The pool of HSF appeared to consist of two cell types, with relatively high (R1) and low (R2) γ-H2AX content (Figure 6A*a1*). Three parameters were determined—the size of R1 fraction (Figure 6A*a2*–*a4*), median values of [m(FL-γH2AX(R2)], and values of m(FL-γH2AX(R2) normalized by the relevant reference (γH2AX index, Figure 6A*a5*,*a6*). In 1 or 3 h after adding hc-cfDNA and sz-cfDNA samples to the culture medium, the number of cells belonging to the R1 fraction (γH2AX,%) increased in all the HSFs. Parameter m(FL-γH2AX(R2) increased in three HSF lines only. After a prolonged HSFs incubation with DNA samples, the γH2AX amount decreased down to the reference.

A positive correlation was revealed between the parameters R1(8-oxodG,%) and R1 (γ-H2AX,%) (Rs = +0.54, *p* = 0.0003, n = 40), Table S1 and Figure 6A*a7.* A quadratic function has appeared to be the best approximation for the link between the two indices of DNA damage degree. The maximum degree of double-strand breaks was fixed at middle levels of the marker 8-oxodG. In order to determine how strongly correlated the levels of the two markers in the cell pools were, we quantified the marker 8-oxodG and the protein γH2AX in the same HSF cells (Figure 6A*a8*) using antibodies labelled with PE (for 8-oxodG) and PB450 (for γH2AX). The analysis of plot distribution in the curve FL-8-oxodG—FL-γH2AX has suggested that the levels of DNA damage markers are positively correlated over the cell pools. There is a minor sub-fraction of cells, however, where the maximum values of the parameter FL- γH2AX can be observed in a small amount of cells simultaneously having relatively low values of the parameter FL-8-oxodG (Figure 6A*a8*).

***PBL***. Hc-cfDNA and sz-cfDNA, but not gDNA samples, stimulated the growth of DNA damage in 1 h of cultivation. In 24 h, the content of the damage marker dropped back below the reference baseline (Figure 6B*b1*–*b3*). The analysis of plot distribution in the FL-8-oxodG—FL-γH2AX curve has shown that, in the same way as HSFs, the peak content of the double-strand break marker can be found in cells with a relatively low content of the oxidation marker (Figure 6B*b4*).

### 2.9. BRCA1 and BRCA2 Expression

**Figure 7:**
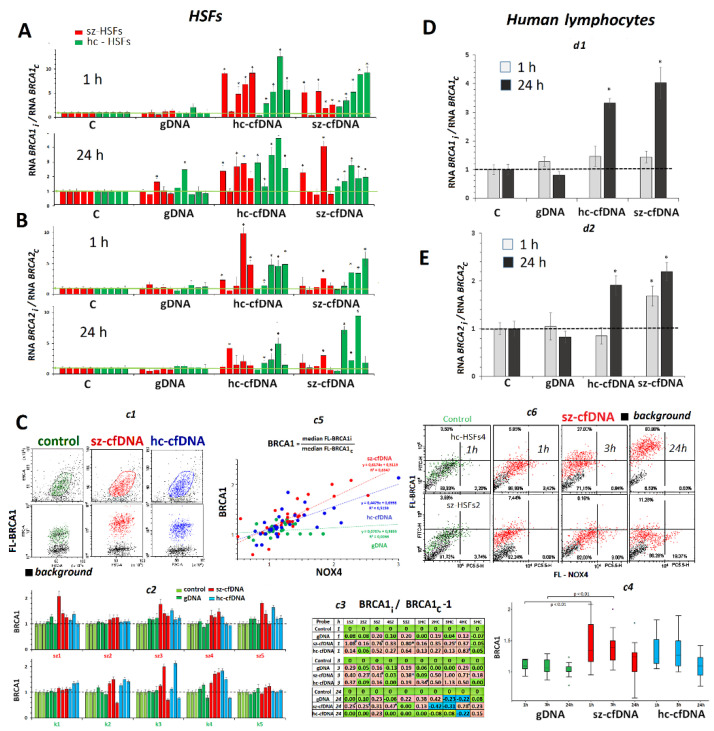
The influence of the DNA samples (50 ng/mL) on the expression of *BRCA1* and *BRCA2* genes in HSFs and PBL. (**A**,**B**). Change of the RNA *BRCA1* and RNA *BRCA2* levels. Average values for three measurements and standard deviation are given. The cultivation time and the DNA samples are indicated on the graph. (*) The response to cfDNA differs from the control HSFs (*p* < 0.01). (**C**). *c1*—The most typical examples (for sz-HSF1,1h) of the BRCA1 assay with FCA in HSFs. *c2*—Index BRCA1: the values of the ratio m(FL-BRCA1)i/m(FL-BRCA1c). Average values for three measurements and standard deviation are given. *c3*—Analysis of relative changes in BRCA1 levels in the presence of DNA samples compared to control. *c4*—Changes in the BRCA1 levels in sample of HSFs (n = 10). *c5*—Dependence of NOX4 index on BRCA1 index. *c6*—sz-HSF2 and hc-HSFs4 staining with two types of antibodies with different labels: NOX4 (PC5.5) and BRCA1 (FITC). (**D**,**E**). Change of the RNA *BRCA1* and RNA *BRCA2* in the PBL in the presence of DNA samples. Average values for four lymphocyte samples and standard deviation are given.

***HSFs.*** In 1 h and 24 h of cultivation, hc-cfDNA and sz-cfDNA samples stimulated an increase of *BRCA1* RNA in nine HSFs (Figure 7A). The *BRCA2* RNA content also increased under the action of cfDNA samples in most HSFs (Figure 7B), demonstrating individual variability.

The cellular amount of BRCA1 protein was measured using FCA (Figure 7C). Hc-cfDNA and sz-cfDNA samples induced similar augmentation of BRCA1 protein content in 1, 3 and 24 h of cultivation in all the HSFs (Figure 7C*c3*,*c4*). The amount of *BRCA1* RNA in 1 and 24 h did not correlate to the amount of BRCA1 protein (Table S1). Perhaps the decrease of *BRCA1* RNA during the first hours after adding cfDNA coupled with the increase of BRCA1 protein can be accounted for by more intense translation of already existing *BRCA1* RNA, while the gene transcription grows later.

We found a positive correlation between relative values of BRCA1 and NOX4 indices for cells that underwent exposure to cfDNA samples (Figure 7C*c5*). The more increased the amount of pro-oxidant protein NOX4 with reference to the control, the higher became the amount of reparation protein BRCA1. No such correlation was observed for gDNA samples.

In order to determine how strongly correlated the levels of BRCA1 and NOX4 proteins are in the cell pool, we performed quantification of the protein contents in the same HSF cell lines (Figure 7C*c6*) using antibodies labelled with PC5.5 (NOX4) or FITC (BRCA1). The analysis of plot distribution in the curve FL-NOX4—FL-BRCA1 has shown that maximum values of the parameter FL-BRCA1 are observed at relatively low values of the parameter FL-NOX4 (Figure 7C*c6*) in the cell pool. Very high intracellular levels of NOX4 protein were associated with relatively low levels of the DNA repair protein BRCA1.

***PBL***. Hc-cfDNA and sz-cfDNA samples, in contrast with gDNA samples, stimulated an approximately equal increase of *BRCA1* RNA and *BRCA2* RNA levels in 24 h of cultivation.

### 2.10. BAX and BCL2 Expression

**Figure 8:**
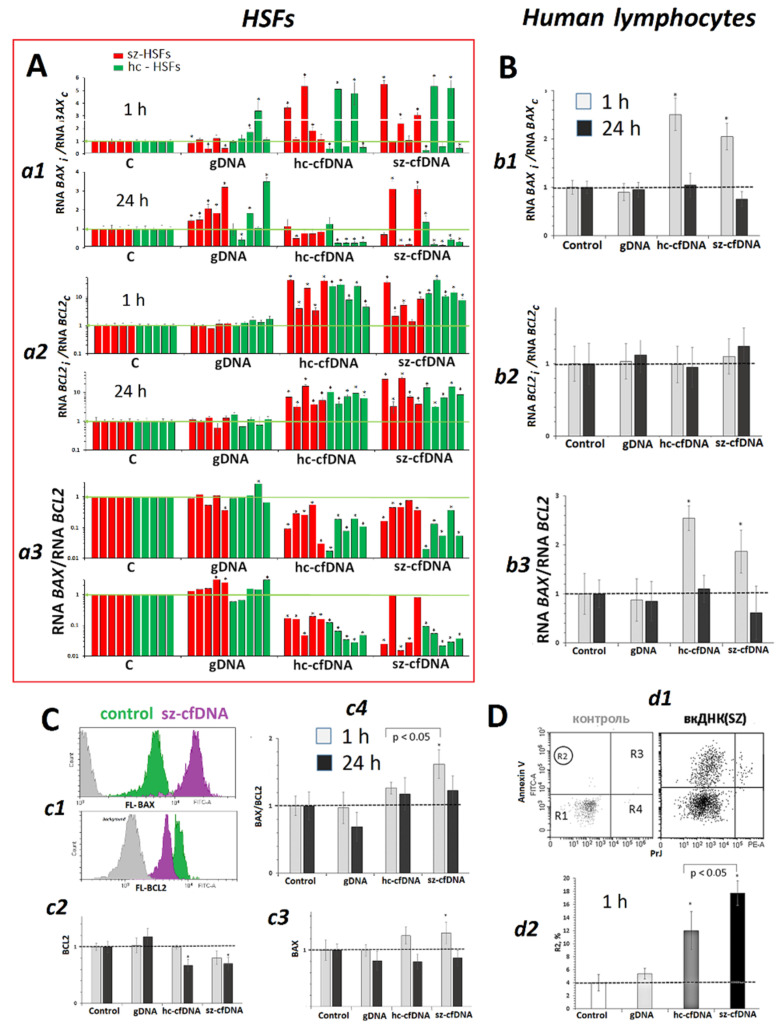
The influence of the DNA samples (50 ng/mL) on the expression of *BCL2* and *BAX* genes in HSFs and PBL. (**A**). *a1*–*a3*—Change of the RNA *BCL2*, RNA *BAX* and the ratio RNA *BAX*/RNA *BCL2* in HSFs. (*) The response to cfDNA differs from the control HSFs (*p* < 0.01). (**B**). *b1*–*b3*—Change of the RNA *BCL2*, RNA *BAX* and the ratio RNA *BAX*/RNA *BCL2* in PBL. (**C**). *c1*—The most typical examples of the BCL2 and BAX assay with FCA in lymphocytes. *c2*–*c4*—Changes in the index BCL2, index BAX and the ratio BAX/BCL2 in the sample of lymphocytes (n = 4). (**D**). *d1*—The most typical example of the apoptosis assay in PBL. *d2*—Proportion of R2 (annexin V+) fraction cells.

***HSFs.*** Hc-cfDNA and sz-cfDNA samples stimulated expression of both *BCL2* and *BAX* genes (Figure 8A*a1*,*a2*). The intracellular amount of *BCL2* RNA in 1 h of cultivation correlated with the amount of *BAX* RNA (Rs = +0.67, *p* < 0.0001, n = 40). A *BAX* RNA/*BCL2* RNA ratio was significantly lower in the cells cultivated with hc-cfDNA and sz-cfDNA than in the reference samples and in the cells cultivated with gDNA (Figure 8A*a3*). This finding may suggest a prevalence of antiapoptotic response in HSFs upon the exposure to hc-cfDNA and sz-cfDNA.

***PBL***. The lymphocyte response to the exposure to DNA samples was very different from the HSF response (Figure 8B). The cfDNA samples had practically no effects on the amount of *BCL2* RNA and stimulated an increase of the amount of *BAX* RNA in 1 h. The *BAX* RNA/*BCL2* RNA ratio increased in 1 h upon exposure to hc-cfDNA and sz-cfDNA (Figure 8B).

Quantification of BCL2 and BAX proteins using FCA confirmed low expression of *BCL2* gene simultaneously with slightly elevated expression of *BAX* gene. The BAX/BCL2 ratio increased in 1 h upon exposure to sz-cfDNA only (Figure 8C*c4*). These findings suggest intensification of apoptosis during the early response of PBL to the action of cfDNAs. We verified the suggestion by estimating the fraction of cells with damaged membrane in PBL pools with the use of the early apoptosis marker annexin V (Figure 8D). In 1 h of cell incubation with cfDNA samples, the fraction of cells with damaged membrane increased from 4% up to 18%, thus corroborating an idea of the transient increase in the fraction of cells with apoptosis signs during early PBL response to the exposure to hc-cfDNA and sz-cfDNA samples.

### 2.11. Correlation Analysis of Changes in the ROS, DNA Damage, RNA and Protein Levels in HSFs

It was of interest to analyze how strong the correlation was between the expression levels of the target genes related to HSF response, changes in the cfDNA profile, ROS content and DNA damage markers. We studied the correlations between parameters showing the cellular early response (1 h) and late response (24 h) separately. The whole group (n = 40) was analyzed, which included 10 control HSF samples and 30 HSFs incubated with gDNA, hc-cfDNA and sz-cfDNA; 10 samples for each DNA type (Table S1).

***ROS (k_dcf_)*.** ROS levels in HSFs in 1 h of cultivation showed a positive correlation with the levels of both RNA and proteins of three genes (*NOX4, HIF1A* and *BRCA1*), as well as the amounts of RNAs transcribed from genes *NFE2L2, SOD1, BRCA2, BCL2* and *BAX.* The maximum coefficient of correlation (Rs) was found for *HIF1A* RNA (Rs = +0.68, *p* < 10^−5^, n = 40), while the minimum was for *BRCA2* RNA (Rs = +0.38, *p* = 0.015, n = 40).

ROS levels in HSFs were positively correlated with oxidation marker R1 (8-oxodG,%) (Rs = 0.41, *p* = 0.009, n = 40) and with the parameters of the marker of double-strand breaks (R1(γH2AX, %), mFL-γH2AX(R2) and γH2AX index) (Rs = 0.50–0.54, *p* < 0.001, n = 40).

***DNA damage*.** Parameter R1 (8-oxodG,%), which indicates the fraction of cells with high DNA oxidation degree, positively correlated with the levels of RNAs transcribed from genes *NOX4, NFE2L2, SOD1, HIF1A, BCL2* and *BAX* (Rs = 0.44–0.54, *p* < 0.005, n = 40) in 1 h of cultivation. In 24 h, correlation with neither gene was observed.

Parameter m(FL-8-oxodG), which indicates oxidation degree in the major cell pool, correlated positively with the amounts of NRF2 and SOD1 proteins and **NOX4** index in 1 h. In 24 h, m(FL-8-oxodG) negatively correlated with RNAs transcribed from *SOD1* and *BAX* genes.

Normalized parameter 8-oxodG index negatively correlated with the amounts of HIF1A and BRCA1 proteins in 24 h.

Parameter R1(γH2AX, %) correlated with the contents of RNAs from genes *NOX4, NFE2L2, SOD1, HIF1A, BRCA1, BCL2* and *BAX* in 1 h. The maximum coefficient of correlation (Rs) was found for *NOX4* gene (Rs = +0.74, *p* <10^−6^, n = 40), while the minimum was for *BRCA1* gene (Rs = +0.58, *p* = 0.0009, n = 40). Positive correlations were also observed between the amounts of γH2AX protein and the amounts of proteins NOX4 and BRCA1. In 24 h, R1(γH2AX, %) negatively correlated with the contents of *HIF1A* RNA and *BCL2* RNA.

Parameter m(FL-γH2AX), which displays the quantity of double-strand breaks in the major cell pool, positively correlated with *NOX4* RNA, m(FL-NOX4) and m(FL-BRCA1) in 1 and 24 h.

***RNAs.*** In 1 h of cultivation, the levels of RNA from all the examined genes correlated between one another, with a single exception of the *BRCA2* RNA–*BAX* RNA pair. Coefficients Rs varied from 0.45 up to 0.82. In 24 h, the level of *NOX4* RNA only correlated with the levels of RNA of all the other genes (except for *BRCA2*). High positive correlations between *SOD1* RNA and *HIF1A* RNA, *HIF1A* RNA and *BCL2* RNA, *BRCA1* RNA and *BCL2* RNA were also retained (Rs = +0.80–0.82, *p* <10^−8^, n = 40).

***Proteins***. Parameter m(FL-BRCA1), which shows the average level of BRCA1 protein in the cell, correlated with the levels of all the proteins analyzed, except for HIF1A, in 1 h. Parameter m(FL- NOX4) correlated with the levels of three of five proteins (γH2AX, NRF2 and BRCA1), and m(FL- NRF2) correlated with the levels of γH2AX, SOD1, NOX4 and BRCA1. The positive correlation between the average levels of the proteins was retained in 24 h.

The NOX4 index, showing changes in NOX4 protein content with reference to the control, positively correlated with the indices γH2AX, SOD1, HIF1A and BRCA1 in 1 h. In 24 h, correlations were observed between NOX4, BRCA1 and NRF2.

## 3. Discussion

In this work, we continued the in vitro study of the bioactivity of cfDNA obtained from the plasma of schizophrenia patients and healthy controls. Earlier, we showed considerable activation of DNA sensors upon the exposure to sz-cfDNA or hc-cfDNA for the HSF [29]. The activation of the sensors launched synthesis of pro-inflammatory cytokines. By the example of model DNA fragments, which contained various numbers of GC-rich and oxidized motifs, it was previously shown that cfDNA could be a potential trigger of adaptive response in the cells and serve as an active agent in the realization of the bystander effect upon the exposure to ionizing radiation [22,30,31,32,33,34,35,36,37,56,57,58]. In this paper, we studied for the first time the ability of real cfDNA samples from human plasma to induce the adaptive response in the cells via the mechanism, which had been earlier described for model DNA fragments only.

In contrast to our previous work [29], we studied the response of two cell types, cultured skin fibroblasts and peripheral blood lymphocytes, to the action of cellular DNA and cfDNA. The aim of the study was the process dynamics and analysis of early and late responses to cfDNA fragments. We therefore used the same cfDNA concentrations (50 ng/mL) for three different time points. It was found earlier that the maximum lymphocyte cell fraction responded to exposure to DNA fragments in the medium at this very concentration [30].

**Figure 9:**
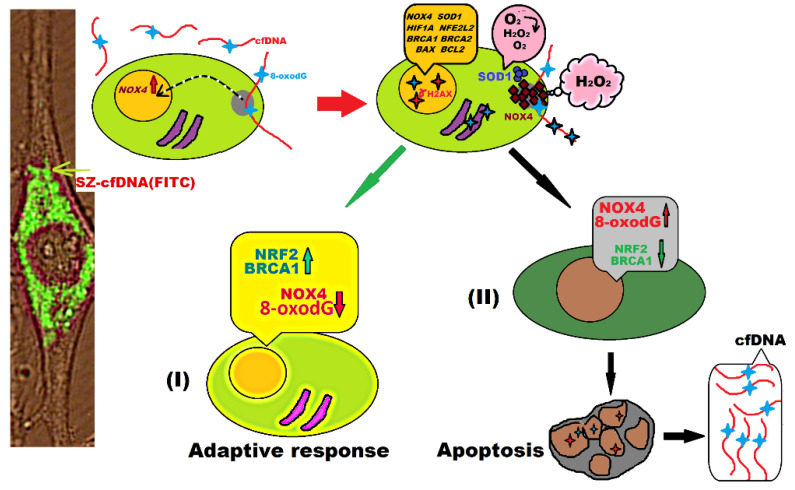
Scheme describing the cell response to the action of cfDNA fragments. A photo from the previous article of a cell interacting with the sz-cfDNA is provided [29].

When adding cfDNA samples to the HSF culture medium, the DNA fragments bound to the cell surface. A small cfDNA fraction penetrated into the cells [29]. The cfDNA fragments were localized in the cytoplasm close to nucleus, in the form of separate granules. Summarizing the experimental facts above, together with the previously published data [29], we could propose a scheme presenting the response of human cells to changes in the characteristics of cfDNA in the medium (Figure 9).

### 3.1. Hc-cfDNA and sz-cfDNA Induced a Transient Oxidative Stress and DNA Damage in HSFs and PBL

The emergence of a cfDNA complex with still unknown structures on the membrane surface led to a significant increase in NOX4 gene expression (Figure 2). An increase in NOX4 expression in response to the exposure to model oxidized DNA fragments was previously observed for MCF7 cancer cells [37], HUVEC endothelial cells [31,56] and mesenchymal stem cells of human adipose tissue [34,35]. We have hypothesized that there are cellular receptors, which interact with oxidized cfDNA regions. The formation of such a complex triggers a signaling pathway, so far undescribed, to launch NOX4 gene expression upon the exposure to oxidized cfDNA fragments.

One can also hypothesize that the complex triggering NOX4 expression includes molecules of the NOX4 protein itself, localized in the cell membrane. We found colocalization of cfDNA fragments and NOX4 protein in HSFs (Figure 2C). The joint localization of cfDNA and NOX4 protein was also found in our earlier study on MCF7 [37].

Simultaneously, with the increase in *NOX4* gene expression, cellular ROS content also became significantly elevated (Figure 1 and Table S1). The ROS content positively correlated with the levels of *NOX4* RNA and NOX4 protein. Previously, we found that ROS production was at a maximum at the sites where cfDNA binds to the cell [57,58], suggesting the role of NOX4, which is also localized at the sites of cfDNA binding to the cell, in creating transient oxidative stress in the cells. The ROS pool appears to include hydrogen peroxide and superoxide anion. This is evidenced by a significant increase in *SOD1* gene transcription, which is proportional to the increase in ROS content. The SOD1 protein transforms superoxide anion into less toxic peroxide and an oxygen molecule. The superoxide anion can be produced by other enzymes of the NOX family, such as the membrane protein NOX2. The ROS burst was of short duration in the cells. Over time, the level of ROS in the cells decreased below the control level (Figure 1B*b2*).

A transient ROS burst leads to cellular DNA damage (Figure 5 and Figure 6). The marker 8-oxodG indicates the oxidation degree of all DNA, including nuclear DNA, mitochondrial DNA, and cfDNA fragments that have penetrated to the cells. The cfDNA fragments connected to the cell surface are eliminated during the preparation of a cell sample for FCA analysis [59]. In our previous study, we have found that the marker 8-oxodG primarily indicates the oxidation degree of mitochondrial DNA [29,37,60]. The number of cells with a high DNA oxidation degree was proportional to the level of ROS in the cells in 1 h (Table S1).

To verify the nuclear DNA damage, we performed quantification of a double-strand break marker, γH2AX protein, which is involved in DNA repair. The amount of γH2AX protein was proportional to the ROS content. A correlation was also found between a fraction of cells with a high oxidation degree (fraction R1(8-oxodG,%)) in the pool and the DNA fragmentation degree (fraction R1(γH2AX,%)) (Table S1).

Thus, exposure of HSFs and PBL to cfDNA induces the early response, which includes mitochondrial and nuclear DNA damage because of a sharp increase in ROS count. With the course of time, the damage degree declines below the control level (Figure 5 and Figure 6).

### 3.2. Hc-cfDNA and sz-cfDNA Induced an Antioxidant Response in HSFs and PBL

In normally functioning cells, oxidative stress results in the induction of an antioxidant response, which aims to reduce the amount of active ROS that damage cellular structures. The antioxidant response in our experiment is indicated by a decrease in ROS content below control values over time.

The pro-oxidant gene NOX4 is known to control the activity of the antioxidant transcription factor NRF2 [41]. In our experiment, we also found a significant correlation between *NOX4* and *NFE2L2* gene expression at the RNA and protein levels in 1 h and in 24 h.

NRF2 has been shown to target a functional ARE motif at the HIF1A locus, revealing a direct regulatory relationship between these two important oxygen-sensing transcription factors [61]. The level of *HIF1A* gene expression increased proportionally to *NFE2L2* gene expression (Table S1), corroborating the findings of those authors. Thus, transient oxidative stress caused by the introduction of hc-cfDNA and sz-cfDNA fragments into the medium stimulates the antioxidant response by synchronously increasing the expression of the genes for two transcription factors—NRF2 and HIF1A.

### 3.3. Hc-cfDNA and sz-cfDNA Induced b HSFs and PBL DNA Damage Response or Apoptosis

Nuclear DNA damage upon the exposure to hc-cfDNA and sz-cfDNA induces a response aimed at enhancing the double-strand break repair processes. This is suggested by a decrease over time in the level of the marker γH2AX in the cells (Figure 6) and an increase in BRCA1 expression, a gene responsible for the repair process (Figure 7, Table S1). Parameters that show the level of expression of these genes in 1 h of cultivation correlated with the parameters indicating ROS content, DNA damage degree, and expression levels of pro-oxidant NOX4 and antioxidant genes.

Simultaneously with the DNA repair process, the expression of *BCL2* anti-apoptotic and *BAX* pro-apoptotic genes was launched (Figure 8). HSFs and PBL differed in the ratio of expression levels of these genes. Under the action of hc-cfDNA and sz-cfDNA on HSFs, the *BAX* RNA/*BCL2* RNA ratio decreased, indicating a prevalence of the antiapoptotic response in the HSF cell pool [54]. Under the action of hc-cfDNA and sz-cfDNA on PBL within an hour, the *BAX* RNA/*BCL2* RNA ratio and the BAX/BCL2 ratio increased, indicating apoptotic processes in the PBL population. The intensification of apoptosis in PBL upon exposure to HSFs hc-cfDNA and sz-cfDNA was confirmed by a test using the marker annexin V, which revealed signs of cell membrane destabilization (Figure 8D). Apoptosis of a small fraction of cells resulted in new fragments of oxidized cfDNA occurring in the medium, which can further induce oxidative stress and the adaptive response in intact cells (Figure 9).

### 3.4. Heterogeneity of HSFs and PBL Pools and HSF Cell Lines by the Type of Their Response to the Action of hc-cfDNA and sz-cfDNA

Analysis of FCA data in Figure 3C,F, Figure 5A*a8*, Figure 6A*a8*, Figure 6B*b4* and Figure 7C*c6* shows heterogeneity of cell pools in terms of response to transient oxidative stress induced by cfDNA fragments. Tentatively, three subpopulations can be distinguished in the cell pool, which were cultivated for 1–3 or 24 h upon exposure to DNA samples. (I)—cells with pronounced signs of an adaptive response. These cells contain plenty of NRF2 and BRCA1 proteins along with a relatively low amount of NOX4 protein and a low level of the DNA oxidation marker. (II)—cells with signs of inefficient repair of DNA damage. The cells contain maximum amounts of NOX4 protein and the marker 8-oxodG, and minimum amounts of antioxidant transcription factor NRF2 and BRCA1 repair protein. (III)—cells that contain medium amounts of the markers.

Obviously, the development of cell response to cfDNA is a dynamic process, and the quantitative ratio between cell subpopulations is constantly changing during the early and late response. In addition, the significant differences in the response of 10 HSF cell lines to the same amounts of hc-cfDNA and sz-cfDNA should be noted. These facts are illustrated, for example, by the data in Figure 7C*c6*.

We did not find any statistically reliable difference in the response of the hc-HSFs and sz-HSFs subgroups to exposure to hc-cfDNA and sz-cfDNA. Variation between strains in the subgroups exceeded variation between the subgroups.

In general terms, the response of HSFs to cfDNA action was correlated with the response of PBL. However, some differences were found. Hc-cfDNA and sz-cfDNA fragments stimulated a stronger oxidative stress associated with higher levels of NOX4 expression, and a stronger antioxidant response and DNA damage response in HSFs. Expression levels of genes for NRF2 and HIF1A factors and *SOD1* and *BRCA1* genes in HSFs increased significantly in 1 h and remained high in 24 h. In PBL, a decrease of *NFE2L2* gene expression upon exposure to hc-cfDNA and sz-cfDNA (Figure 3) and a 24-h delayed increase of *HIF1A*, *SOD1* and *BRCA1* gene expression were observed (Figure 4 and Figure 7). A temporary arrest of the expression and activity of the NRF2 protein was also registered earlier in the cells’ response to various environmental factors [62]. We believe that this process is necessary to ensure a sufficiently high ROS content, which is necessary for the development of an adaptive response [30]. Despite the lower ROS content, apoptosis is more activated in lymphocytes during the first hours of exposure to cfDNA fragments (Figure 8). A significant reduction in the number of cells with damaged DNA in PBL in 24 h can apparently be explained by the elimination of apoptotic cells from the cell pool.

We found no considerable difference in the bioactivity of sz-cfDNA and hc-cfDNA samples with respect to HSFs or PBL. A significantly stronger response of HSFs and PBL to hc-cfDNA and sz-cfDNA compared to gDNA may be due to a higher oxidation degree of sz-cfDNA and hc-cfDNA. Moreover, a set of sequences that are altered in cfDNA compared to cellular DNA, different methylation rates, and various (along with 8-oxodG) oxidative base modifications may matter.

In spite of the fact that sz-cfDNA and hc-cfDNA samples at the same concentration, demonstrated approximately the same bioactivity in vitro with respect to HSFs and PBL, the situation may be different in the body. The biological activity of cfDNA significantly depends on the content of active fragments in the intercellular medium [63,64]. The concentration of common cfDNA fragments and the concentration of oxidized and easily oxidized GC-rich fragments in SZ blood were several times higher than those in healthy control subjects [16,17]. The elevated content of biologically active cfDNA in SZ patients can significantly intensify its action on the body cells, stimulating an oxidative stress and an adaptive response. In order to test this assumption, a future examination of cell exposure to various doses of cfDNA for the same period of time is planned, although oxidative conditions in vitro and in vivo are too different to extrapolate the concentrations directly.

**Conclusion.** In human cell cultures, hc-cfDNA and sz-cfDNA stimulate a ROS-mediated adaptive response. The mediating ROS are produced mostly by NOX4 and SOD1 enzymes and launch the antioxidant, repair and antiapoptotic cytoprotective processes.

## 4. Materials and Methods

### 4.1. Experimental Design and Participants

Circulating cfDNA was isolated from the plasma of schizophrenia subjects (sz-cfDNA) and healthy controls (hc-cfDNA). Cellular DNA (gDNA) was extracted from leukocytes of healthy controls. Healthy male volunteers (N = 10) aged 21 to 35 years and unmedicated schizophrenia patients (N = 10) aged 18 to 35 years (27 ± 8 years) participated in the study. A detailed description of the study participants and characteristics of DNA samples was presented in the previous article (Part 1) [29]. DNA was isolated from blood plasma and leukocytes using extraction with organic solvents.

We studied the responses of two cell types to exposure to three DNA samples (gDNA, hc-cfDNA, sz-cfDNA): cultured human skin fibroblasts derived from five schizophrenia patients (sz-HSF1-5) and five healthy controls (hc-HSF1-5), and PBMC from the blood of four controls. DNA samples in an amount of 50 ng/mL were introduced into the culture medium at hours 1 and 3 (early response) and hour 24 (late response).

In 1 and 24 h, changes in the contents of RNA for eight genes (*NOX4, NFE2L2, SOD1, HIF1A, BRCA1, BRCA2, BAX* and *BCL2*) were registered and analyzed. In 1, 3 and 24 h, changes in the contents of six proteins (NOX4, NRF2, SOD1, HIF1A, γH2AX and BRCA1) and the oxidation marker 8-oxodG were also registered and analyzed.

### 4.2. Cell Cultures

#### 4.2.1. Human Skin Fibroblasts (HSFs)

Primary adult HSFs of healthy controls (n = 5) and SZ patients (n = 5) were obtained from the collection of the Research Centre for Medical Genetics. HSFs were subcultured with 10% serum four or fewer times before the experiments. The fibroblasts were grown in a medium complemented with 10% serum until subconfluency was reached. To study the cells’ response to changes in the properties of cfDNA, the cells were transferred to a ‘Hybris’ serum-free medium consisting of the basic medium and a serum-free supplement for 24 h. The supplement contained purified human albumin and a growth factor cocktail (‘Paneco’, Moscow, Russia). Various DNA samples were added at 50 ng/mL to the cell culture medium for 1 h, 3 h and 24 h, in order to stimulate changes in the properties of cfDNA. Each sample was assayed in triplicate.

#### 4.2.2. Peripheral Blood Lymphocyte (PBL)

The blood samples were collected from four healthy volunteers on the day of the experiment. Each 15 mL sample was drawn under strict aseptic conditions from a peripheral vein using a syringe flushed with heparin. The total pool of peripheral blood mononuclear cells (PBMC) was centrifugally segregated at 1500 rpm for 30 min using lymphocyte separation media (Histopaque 1077, 1.077 g/mL, ‘Sigma’, St. Louis, MO, USA). After isolation, the PBM cells (10^6^/mL) were cultured for 3 h at 37 °C in the medium containing 1 mMol/L HEPES (‘Fluka’, Buchs, Switzerland), Hank’s solution and 10% embryonic calf serum (‘HyClone’, Logan, UT, USA). Next, DNA samples were added to the culture medium and incubated for 1 h, 3 h or 24 h.

### 4.3. Ethical Approval for Operating with Cultured and Primary (Blood Leukocytes) Human Cells

The study was conducted in conformity with the latest version of the Declaration of Helsinki and approved by the Independent Interdisciplinary Ethics Committee on Ethical Review for Clinical Studies (Protocol #4 as of 15 March 2019 for the scientific minimally invasive study “Molecular and neurophysiological markers of endogenous human psychoses”). Each participant signed an informed consent for participation in and publication of the anonymized findings of the study after the procedures had been clearly explained.

### 4.4. Synthesis of Fluorescently Labeled sz-cfDNA Probe

In order to prepare the FL-DNA (FITC) probe, a set of reagents (Label IT^®^ Nucleic Acid Labeling Kits, Fluorescein (MIR 3200), ‘Gamma Biosciences’, Menlo Park, CA, USA) was used, which interacts directly with the DNA chemical groups.

### 4.5. ROS Assay on HSFs

Total fluorescence assay was applied to analyze the HSFs in the 96-well plate format at λ_ex_ = 488 nm and λ_em_ = 528 nm (EnSpire equipment, ‘PerkinElmer’, Turku, Finland). The medium was replaced with 5 μm H2DCFH-DA (Molecular Probes/Invitrogen, ‘Thermo Fisher Scientific’, Waltham, MA, USA) in a PBS solution, and a fluorescence intensity splash was registered at 37 °C. Eight (4 × 2) repeated measurements were taken for each DNA sample and 16 for the control. I−time coordinates were used in order to present the graphs. The slope of the line (kdcf), which reflects the dependence of the fluorescence intensity on time, characterizes the level of ROS in HSFs.

### 4.6. Flow Cytometry Analysis (FCA)

#### 4.6.1. Cell Cultivation

HSFs were grown for 1 h, 3 h or 24 h in 60 mm dishes, where various DNA probes could be added. Prior to FCA, cells were washed with Versene solution and treated with 0.25% trypsin under light microscope observation. Then the cells were transferred to Eppendorf tubes, washed with culture medium, centrifuged and resuspended in PBS. PBL were cultured for 1 h or 24 h in 60 mm dishes, where various DNA probes could be added. Cells were washed with PBS, centrifuged and resuspended in PBS.

#### 4.6.2. Fixation and Quantification

Paraformaldehyde (Sigma, St. Louis, MO, USA) was added at a final concentration of 3% at 37 °C for 10 min to fix the cells, which were then washed three times with 0.5% BSA-PBS and permeabilized with 0.1% Triton X-100 (Sigma, St. Louis, MO, USA) in PBS. Cells (∼50 × 10^3^) were washed with 0.5% BSA-PBS and stained with antibodies: NRF2-FITC(bs1074r-fitc, ‘Bioss. Inc. ’, Boston, MA, USA), 8-oxodG-PE (sc-393871 PE, ‘Santa Cruz Biotechnology, Inc. ’, Santa Cruz, CA, USA), NOX4 -PC5.5 (bs-1091r-cy5-5, ‘Bioss. Inc. ’, Boston, MA, USA) or NOX4-FITC (Q9NPH5, ‘Cusabio Technology’, Houston, TX, USA), BRCA1-FITC (NB100-598F, ‘Novus Biologicals, LLC’, Centennial, CO, USA), γH2AX-pb450 (nb100-384AF405, ‘Novus Biologicals, LLC’, Centennial, CO, USA), SOD-PE (sc-11407 and rabbit IgG-PE sc-3753, ‘Santa Cruz Biotechnology, Inc. ’, Santa Cruz, CA, USA), or HIF1A-PE (NB100-479, ‘Novus Biologicals, LLC’, Centennial, CO, USA and rabbit IgG-PE sc-3753, ‘Santa Cruz Biotechnology, Inc.’, Santa Cruz, CA, USA). To quantify the background fluorescence, a fraction of the cells was stained with secondary FITC (PE)-conjugated antibodies only. The cells were assayed at CytoFLEX S (‘Beckman Coulter’, Indianapolis, IN, USA).

#### 4.6.3. PBL ROS Assay 

PBL were washed with PBS and incubated with 10 μM H2DCFH-DA (Invitrogen, ‘Thermo Fisher Scientific’, Waltham, MA, USA) in the dark for 20 min at 37 °C. After washing with PBS, the cells were immediately analyzed by FCA.

### 4.7. Real-Time PCR Assay

Isolation of total mRNA was performed using a RNeasy Mini kit (‘Qiagen’, Hilden, Germany). RNA was quantified using a Quant-iT RiboGreen RNA reagent dye (‘MoBiTec’, Göttingen, Germany) on a tablet reader (EnSpire Equipment, ‘PerkinElmer’, Turku, Finland) at λ_em_ = 487 nm, λ_fl_ = 524 nm. After DNAse I treatment, RNA samples were reverse transcribed using a Reverse Transcriptase kit (‘Sileks’, Moscow, Russia). PCR was conducted with the specific primers and Sybr-Green intercalating dye on a StepOnePlus device (‘Applied Biosystems’, Foster City, CA, USA). The primers were selected and synthesized by ‘Evrogen’ (Moscow, Russia). The internal standard was TBP gene.

The PCR reaction mixture in a volume of 25 µL consisted of 2.5 µL PCR buffer (700 mMol/L Tris-HCl, pH 8.6); 166 mMol/L ammonium sulfate, 35 mMol/L MgCl_2_, 2 µL 1.5 mMol/L dNTP solution; and 1 µL 30 pMol/L primer solution, cDNA. PCR conditions were chosen individually for each primer pair. After denaturation for 4 min at 95 °C, 40 amplification cycles were performed in the following order: 94 °C for 20 s, 56–62 °C for 30 s, 72 °C for 30 s, and 72 °C for 5 min. The data were processed using a calibration plot with a resultant error of 2%.

### 4.8. Fluorescence Microscopy

An Axio Scope.A1 microscope (‘Carl Zeiss’, Oberkochen, Germany) was applied.

A Sz-cfDNA probe was added to the medium for 1 h at 50 ng/mL. Cells were washed with PBS three times, fixed for 20 min in 3% paraformaldehyde at 4 °C, washed with PBS and incubated for 4 h at 4 °C with PC5.5 –NOX4 antibodies. Previously washed with 0.01% Triton X-100 in PBS, HSFs were then washed with PBS and stained with 2 μg/mL DAPI.

### 4.9. Statistical Data Processing

The tests were repeated in triplicate. In FCA, the medians of the signal intensities were analyzed. Non-parametric Mann–Whitney U-test was applied to determine the significance of the observed differences. *p*-values < 0.01 were considered statistically significant. Data processing was performed with Excel, Microsoft Office (‘Microsoft’, Redmond, WA, USA) and StatPlus2007 Professional software (http://www.analystsoft.com accessed on 1 December 2022).

## Supplementary Materials

The following supporting information can be downloaded at https://www.mdpi.com/article/10.3390/genes13122283/s1; Table S1: Spearman’s rank correlation (Rs and p-value) for the RNAs, proteins and 8-oxodG levels in HSFs incubated with DNA samples. The analysis was performed for the entire group (n = 40), which included 10 control samples and 30 samples of HSFs exposed to gDNA, hc-cfDNA and sz-cfDNA, 10 samples for each sample type.
