# Supplementary material for: In Vitro Analysis of Biological Activity of Circulating Cell-Free DNA Isolated from Blood Plasma of Schizophrenic Patients and Healthy Controls—Part 2: Adaptive Response
Source: Genes (Basel). 2022 Dec 4;13(12):2283. doi: 10.3390/genes13122283 (PMC9777734; doi:10.3390/genes13122283)
Supplement: Supplementary file 1 [file genes-13-02283-s001.zip › genes-2050806-supplementary.pdf]

Table S1. Spearman's rank correlation (Rs and p-value) for the RNAs, proteins and 8-oxodG levels in HSFs incubated with DNA samples. The analysis was performed for the entire group (n=40), which included 10 control samples and 30 samples of HSFs exposed to gDNA, hc-cfDNA and sz-cfDNA, 10 samples for each sample type.

1h		ROS	NOX4			8-oxodG			γH2AX			NRF2			SOD1			HIF1A			BRCA1			*BRCA2*	*BAX1*	*BCL2*
		(k_{DCF})	RNA	m	Index	R1 %	m	Index	R1 %	m	Index	RNA	m	Index	RNA	m	Index	RNA	m	Index	RNA	m	Index	RNA	RNA	RNA
ROS	(k_{DCF})		0.53	0.43	0.37	0.41	0.1	0.1	0.50	0.52	0.54	0.64	0.2	0.1	0.66	0.0	0.1	0.68	0.45	0.2	0.54	0.43	0.37	0.38	0.51	0.55
			$5\cdot10^{-4}$	0.005	0.02	0.009	0.5	0.5	0.001	$8\cdot10^{-4}$	$3\cdot10^{-4}$	$1\cdot10^{-5}$	0.2	0.3	$4\cdot10^{-6}$	0.8	0.7	$3\cdot10^{-6}$	0.004	0.1	$3\cdot10^{-4}$	0.005	0.02	0.015	$9\cdot10^{-4}$	$2\cdot10^{-4}$
NOX4	RNA	0.53		0.48	0.50	0.54	0.2	-0.1	0.74	0.41	0.0	0.80	0.2	0.2	0.81	0.0	0.2	0.80	0.1	0.55	0.71	0.59	0.54	0.45	0.72	0.81
		$5\cdot10^{-4}$		0.002	0.001	$3\cdot10^{-4}$	0.3	0.4	$1\cdot10^{-7}$	0.009	0.8	$1\cdot10^{-9}$	0.1	0.1	$1\cdot10^{-10}$	0.9	0.3	$1\cdot10^{-9}$	0.7	$2\cdot10^{-4}$	$2\cdot10^{-7}$	$1\cdot10^{-4}$	$3\cdot10^{-4}$	0.004	$2\cdot10^{-7}$	$1\cdot10^{-10}$
	m	0.43	0.48		0.1	0.2	-0.0	-0.2	0.42	0.62	0.1	0.3	0.48	0.0	0.42	0.3	-0.0	0.42	0.2	0.1	0.2	0.74	0.3	-0.2	0.3	0.3
		0.005	0.002		0.3	0.1	0.8	0.3	0.007	0.002	0.4	0.1	0.002	0.8	0.007	0.1	0.9	0.007	0.3	0.4	0.2	$1\cdot10^{-7}$	0.1	0.1	0.1	0.1
	Index	0.37	0.50	0.1		0.43	0.32	0.31	0.62	0.1	0.46	0.66	0.1	0.0	0.60	0.2	0.52	0.46	0.2	0.60	0.44	0.2	0.80	0.1	0.60	0.54
		0.02	0.001	0.3		0.005	0.04	0.04	$2\cdot10^{-5}$	0.7	0.002	$4\cdot10^{-6}$	0.7	0.8	$1\cdot10^{-4}$	0.2	$8\cdot10^{-4}$	0.002	0.1	$1\cdot10^{-4}$	0.004	0.2	$1\cdot10^{-9}$	0.4	$1\cdot10^{-4}$	$3\cdot10^{-4}$
8-oxodG	R1 %	0.41	0.54	0.2	0.43		0.45	0.45	0.60	0.2	0.1	0.44	0.2	0.0	0.44	0.1	0.2	0.52	-0.0	0.44	0.2	0.37	0.48	0.0	0.48	0.45
		0.009	$3\cdot10^{-4}$	0.1	0.005		0.004	0.004	$1\cdot10^{-4}$	0.3	0.8	0.004	0.2	0.9	0.004	0.5	0.3	$8\cdot10^{-4}$	0.8	0.004	0.3	0.02	0.002	0.8	0.002	0.004
	m	0.1	0.2	-0.0	0.32	0.45		0.70	0.2	-0.0	0.0	-0.0	0.35	-0.1	0.0	0.66	0.1	-0.0	0.1	0.1	-0.2	0.2	0.2	-0.1	0.3	-0.0
		0.5	0.3	0.8	0.04	0.004		$5\cdot10^{-7}$	0.1	0.9	0.9	0.9	0.03	0.7	0.8	$4\cdot10^{-6}$	0.8	0.9	0.8	0.6	0.2	0.2	0.2	0.4	0.1	0.9
	Index	0.1	-0.1	-0.2	0.31	0.45	0.70		-0.1	-0.1	0.1	0.1	0.2	0.2	0.2	0.3	-0.1	0.2	0.1	-0.1	-0.1	-0.0	0.2	0.3	0.2	-0.0
		0.5	0.4	0.3	0.04	0.004	$5\cdot10^{-7}$		0.4	0.4	0.7	0.7	0.2	0.2	0.3	0.1	0.5	0.2	0.4	0.6	0.6	0.9	0.3	0.1	0.3	0.8
γH2AX	R1 %	0.50	0.74	0.42	0.62	0.60	0.2	-0.1		0.2	0.1	0.62	0.3	0.1	0.60	0.2	0.2	0.58	0.1	0.62	0.51	0.58	0.64	0.1	0.71	0.60
		0.001	$1\cdot10^{-7}$	0.007	$2\cdot10^{-5}$	$1\cdot10^{-4}$	0.1	0.4		0.2	0.7	$2\cdot10^{-5}$	0.1	0.4	$4\cdot10^{-5}$	0.1	0.1	$8\cdot10^{-5}$	0.3	$2\cdot10^{-5}$	$9\cdot10^{-4}$	$1\cdot10^{-4}$	$1\cdot10^{-5}$	0.5	$5\cdot10^{-7}$	$4\cdot10^{-5}$
	m	0.52	0.41	0.62	0.1	0.2	-0.0	-0.1	0.2		0.68	0.2	0.31	-0.1	0.3	-0.1	0.2	0.2	0.3	-0.2	0.2	0.64	-0.1	-0.1	0.1	0.1
		$8\cdot10^{-4}$	0.009	0.002	0.7	0.3	0.9	0.4	0.2		$3\cdot10^{-6}$	0.1	0.04	0.5	0.2	0.4	0.3	0.1	0.1	0.3	0.1	$1\cdot10^{-5}$	0.5	0.6	0.4	0.5
	Index	0.54	0.0	0.1	0.46	0.1	0.0	0.1	0.1	0.68		0.48	-0.1	0.1	0.3	-0.1	-0.1	0.3	0.2	-0.1	0.1	0.1	0.1	0.1	0.0	-0.0
		$3\cdot10^{-4}$	0.8	0.4	0.002	0.8	0.9	0.7	0.7	$3\cdot10^{-6}$		0.002	0.7	0.6	0.1	0.6	0.6	0.1	0.2	0.4	0.6	0.6	0.4	0.6	0.8	0.8
NRF2	RNA	0.64	0.80	0.3	0.66	0.44	-0.0	0.1	0.62	0.2	0.48		0.1	0.3	0.80	0.0	0.3	0.74	0.0	0.47	0.80	0.37	0.58	0.60	0.66	0.74
		$1\cdot10^{-5}$	$1\cdot10^{-9}$	0.1	$4\cdot10^{-6}$	0.004	0.9	0.7	$2\cdot10^{-5}$	0.1	0.002		0.6	0.1	$1\cdot10^{-9}$	0.9	0.1	$1\cdot10^{-7}$	0.8	0.002	$1\cdot10^{-9}$	0.02	$1\cdot10^{-4}$	$4\cdot10^{-5}$	$4\cdot10^{-6}$	$1\cdot10^{-7}$
	m	0.2	0.2	0.48	0.1	0.2	0.35	0.2	0.3	0.31	-0.1	0.1		0.0	0.1	0.50	-0.2	0.1	0.2	-0.1	0.1	0.60	-0.0	-0.2	0.1	0.1
		0.2	0.1	0.002	0.7	0.2	0.03	0.2	0.1	0.04	0.7	0.6		0.8	0.7	0.001	0.1	0.4	0.3	0.5	0.7	$4\cdot10^{-5}$	0.9	0.3	0.5	0.6
	Index	0.1	0.2	0.0	0.0	0.0	-0.1	0.2	0.1	-0.1	0.1	0.3	0.0		0.1	-0.3	0.0	0.2	0.1	0.1	-0.3	-0.1	0.2	0.53	-0.2	0.1
		0.3	0.1	0.8	0.8	0.9	0.7	0.2	0.4	0.5	0.6	0.1	0.8		0.4	0.1	0.9	0.1	0.6	0.5	0.1	0.5	0.1	$4\cdot10^{-4}$	0.3	0.7
SOD1	RNA	0.66	0.81	0.42	0.60	0.44	0.0	0.2	0.60	0.3	0.3	0.80	0.1	0.1		0.0	0.2	0.81	-0.0	0.48	0.74	0.52	0.51	0.47	0.74	0.81
		$4\cdot10^{-6}$	$1\cdot10^{-10}$	0.007	$1\cdot10^{-4}$	0.004	0.8	0.3	$4\cdot10^{-5}$	0.2	0.1	$1\cdot10^{-9}$	0.7	0.4		0.9	0.1	$1\cdot10^{-10}$	0.9	0.002	$1\cdot10^{-7}$	$8\cdot10^{-4}$	$9\cdot10^{-4}$	0.002	$1\cdot10^{-7}$	$1\cdot10^{-10}$
	m	0.0	0.0	0.3	0.2	0.1	0.66	0.3	0.2	-0.1	-0.1	0.0	0.50	-0.3	0.0		0.1	-0.1	0.2	0.2	0.0	0.40	0.0	-0.2	0.3	0.0
		0.8	0.9	0.1	0.2	0.5	$4\cdot10^{-6}$	0.1	0.1	0.4	0.6	0.9	0.001	0.1	0.9		0.3	0.7	0.2	0.2	0.9	0.01	0.9	0.1	0.1	0.8
	Index	0.1	0.2	-0.0	0.52	0.2	0.1	-0.1	0.2	0.2	-0.1	0.3	-0.2	0.0	0.2	0.1		0.1	-0.55	-0.1	0.2	-0.0	0.55	0.0	0.3	0.3
		0.7	0.3	0.9	$8\cdot10^{-4}$	0.3	0.8	0.5	0.1	0.3	0.6	0.1	0.1	0.9	0.1	0.3		0.3	$2\cdot10^{-4}$	0.7	0.2	0.9	$2\cdot10^{-4}$	0.9	0.1	0.1
HIF1A	RNA	0.68	0.80	0.42	0.46	0.52	-0.0	0.2	0.58	0.2	0.3	0.74	0.1	0.2	0.81	-0.1	0.1		0.31	0.48	0.68	0.51	0.40	0.49	0.71	0.82
		$3\cdot10^{-6}$	$1\cdot10^{-9}$	0.007	0.002	$8\cdot10^{-4}$	0.9	0.2	$8\cdot10^{-5}$	0.1	0.1	$1\cdot10^{-7}$	0.4	0.1	$1\cdot10^{-10}$	0.7	0.3		0.04	0.002	$3\cdot10^{-6}$	$9\cdot10^{-4}$	0.008	0.001	$5\cdot10^{-7}$	$1\cdot10^{-10}$
	m	0.45	0.1	0.2	0.2	-0.0	0.1	0.1	0.1	0.3	0.2	0.0	0.2	0.1	-0.0	0.2	-0.55	0.31		0.43	0.2	0.3	0.3	0.2	0.1	0.1
		0.004	0.7	0.3	0.1	0.8	0.8	0.4	0.3	0.1	0.2	0.8	0.3	0.6	0.9	0.2	$2\cdot10^{-4}$	0.04		0.005	0.3	0.1	0.1	0.2	0.3	0.5
	Index	0.2	0.55	0.1	0.60	0.44	0.1	-0.1	0.62	-0.2	-0.1	0.47	-0.1	0.1	0.48	0.2	-0.1	0.48	0.43		0.39	0.43	0.56	0.1	0.55	0.44
		0.1	$2\cdot10^{-4}$	0.4	$1\cdot10^{-4}$	0.004	0.6	0.6	$2\cdot10^{-5}$	0.3	0.4	0.002	0.5	0.5	0.002	0.2	0.7	0.002	0.005		0.01	0.005	$1\cdot10^{-4}$	0.5	$1\cdot10^{-4}$	0.003
BRCA1	RNA	0.54	0.71	0.2	0.44	0.2	-0.2	-0.1	0.51	0.2	0.1	0.80	0.1	-0.3	0.74	0.0	0.2	0.68	0.2	0.39		0.3	0.37	0.74	0.54	0.80
		$3\cdot10^{-4}$	$2\cdot10^{-7}$	0.2	0.004	0.3	0.2	0.6	$9\cdot10^{-4}$	0.1	0.6	$1\cdot10^{-9}$	0.7	0.1	$1\cdot10^{-7}$	0.9	0.2	$3\cdot10^{-6}$	0.3	0.01		0.1	0.02	$1\cdot10^{-7}$	$3\cdot10^{-4}$	$1\cdot10^{-9}$
	m	0.43	0.59	0.74	0.2	0.37	0.2	-0.0	0.58	0.64	0.1	0.37	0.60	-0.1	0.52	0.40	-0.0	0.51	0.3	0.43	0.3		0.40	-0.2	0.56	0.52
		0.005	$1\cdot10^{-4}$	$1\cdot10^{-7}$	0.2	0.02	0.2	0.9	$1\cdot10^{-4}$	$1\cdot10^{-5}$	0.6	0.02	$4\cdot10^{-5}$	0.5	$8\cdot10^{-4}$	0.01	0.9	$9\cdot10^{-4}$	0.1	0.005	0.1		0.011	0.3	$1\cdot10^{-4}$	$8\cdot10^{-4}$
	Index	0.37	0.54	0.3	0.80	0.48	0.2	0.2	0.64	-0.1	0.1	0.58	-0.0	0.2	0.51	0.0	0.55	0.40	0.3	0.56	0.37	0.40		0.1	0.53	0.52
		0.02	$3\cdot10^{-4}$	0.1	$1\cdot10^{-9}$	0.002	0.2	0.3	$1\cdot10^{-5}$	0.5	0.4	$1\cdot10^{-4}$	0.9	0.1	$9\cdot10^{-4}$	0.9	$2\cdot10^{-4}$	0.008	0.1	$1\cdot10^{-4}$	0.02	0.011		0.3	$1\cdot10^{-4}$	$8\cdot10^{-4}$
BRCA2	RNA	0.38	0.45	-0.2	0.1	0.0	-0.1	0.3	0.1	-0.1	0.1	0.60	-0.2	0.53	0.47	-0.2	0.0	0.49	0.2	0.1	0.74	-0.2	0.1		-0.1	0.52
		0.015	0.004	0.1	0.4	0.8	0.4	0.1	0.5	0.6	0.6	$4\cdot10^{-5}$	0.3	$4\cdot10^{-4}$	0.002	0.1	0.9	0.001	0.2	0.5	$1\cdot10^{-7}$	0.3	0.3		0.7	$8\cdot10^{-4}$
BAX	RNA	0.51	0.72	0.3	0.60	0.48	0.3	0.2	0.71	0.1	0.0	0.66	0.1	-0.2	0.74	0.3	0.3	0.71	0.1	0.55	0.54	0.56	0.53	-0.1		0.71
		$9\cdot10^{-4}$	$2\cdot10^{-7}$	0.1	$1\cdot10^{-4}$	0.002	0.1	0.3	$5\cdot10^{-7}$	0.4	0.8	$4\cdot10^{-6}$	0.5	0.3	$1\cdot10^{-7}$	0.1	0.1	$5\cdot10^{-7}$	0.3	$1\cdot10^{-4}$	$3\cdot10^{-4}$	$1\cdot10^{-4}$	$1\cdot10^{-4}$	0.7		$5\cdot10^{-7}$
BCL2	RNA	0.55	0.81	0.3	0.54	0.45	-0.0	-0.0	0.60	0.1	-0.0	0.74	0.1	0.1	0.81	0.0	0.3	0.82	0.1	0.44	0.80	0.52	0.52	0.52	0.71	
		$2\cdot10^{-4}$	$1\cdot10^{-10}$	0.1	$3\cdot10^{-4}$	0.004	0.9	0.8	$4\cdot10^{-5}$	0.5	0.8	$1\cdot10^{-7}$	0.6	0.7	$1\cdot10^{-10}$	0.8	0.1	$1\cdot10^{-10}$	0.5	0.003	$1\cdot10^{-9}$	$8\cdot10^{-4}$	$8\cdot10^{-4}$	$8\cdot10^{-4}$	$5\cdot10^{-7}$	

24 h		NOX4			8-oxodG			γH2AX			NRF2			SOD1			HIF1A			BRCA1			*BRCA2*	*BAX1*	*BCL2*
		RNA	m	Index	R1 %	m	Index	R1 %	m	Index	RNA	m	Index	RNA	m	Index	RNA	m	Index	RNA	m	Index	RNA	RNA	RNA
NOX4	RNA		0.1<br>0.6	0.2<br>0.2	0.1<br>0.6	-0.1<br>0.6	0.0<br>0.9	-0.2<br>0.2	0.0<br>0.9	-0.0<br>0.9	0.78<br>$3\cdot10^{-9}$	-0.0<br>0.7	-0.0<br>1.0	0.47<br>0.002	0.1<br>0.5	0.1<br>0.5	0.51<br>$9\cdot10^{-4}$	-0.1<br>0.6	-0.0<br>0.8	0.60<br>$4\cdot10^{-5}$	0.1<br>0.6	0.54<br>$3\cdot10^{-4}$	0.64<br>$1\cdot10^{-5}$	-0.0<br>0.8	0.71<br>$2\cdot10^{-7}$
	m	0.1<br>0.6		0.42<br>0.006	-0.0<br>0.9	0.35<br>0.03	-0.34<br>0.03	0.32<br>0.04	0.58<br>$1\cdot10^{-4}$	0.0<br>0.9	-0.0<br>0.8	0.64<br>$1\cdot10^{-5}$	0.54<br>$3\cdot10^{-4}$	0.0<br>0.9	-0.1<br>0.6	-0.0<br>0.7	0.42<br>0.006	0.56<br>$2\cdot10^{-4}$	-0.0<br>0.8	0.0<br>0.9	0.80<br>$1\cdot10^{-9}$	0.42<br>0.006	0.1<br>0.5	-0.1<br>0.6	0.0<br>0.9
	Index	0.2<br>0.2	0.42<br>0.006		-0.1<br>0.6	0.1<br>0.6	0.1<br>0.5	-0.0<br>0.8	-0.1<br>0.6	0.1<br>0.4	-0.0<br>0.7	0.1<br>0.4	0.50<br>0.001	-0.0<br>0.9	-0.0<br>0.8	0.1<br>0.4	0.3<br>0.1	0.1<br>0.7	-0.0<br>0.8	-0.0<br>0.9	0.1<br>0.4	0.50<br>0.001	0.3<br>0.1	0.1<br>0.4	-0.0<br>0.8
8-oxodG	R1 %	0.1<br>0.6	-0.0<br>0.9	-0.1<br>0.6		0.3<br>0.1	0.2<br>0.3	0.0<br>0.8	0.1<br>0.4	0.2<br>0.3	0.0<br>1.0	0.0<br>0.9	-0.1<br>0.6	0.0<br>0.9	0.1<br>0.5	0.1<br>0.5	0.1<br>0.4	0.2<br>0.3	0.1<br>0.7	0.2<br>0.3	0.0<br>0.9	-0.1<br>0.7	0.1<br>0.1	0.1<br>0.5	0.2<br>0.2
	m	-0.1<br>0.6	0.35<br>0.03	0.1<br>0.6	0.3<br>0.1		0.2<br>0.3	0.2<br>0.2	0.2<br>0.3	0.2<br>0.2	-0.2<br>0.3	0.1<br>0.7	0.1<br>0.5	-0.40<br>0.011	0.1<br>0.4	0.1<br>0.5	-0.1<br>0.4	-0.46<br>0.002	0.1<br>0.7	-0.1<br>0.6	0.3<br>0.1	-0.1<br>0.7	-0.0<br>0.9	-0.42<br>0.007	0.1<br>0.6
	Index	0.0<br>0.9	-0.34<br>0.03	0.1<br>0.5	0.2<br>0.3	0.2<br>0.3		-0.38<br>0.015	-0.2<br>0.2	-0.0<br>0.9	-0.2<br>0.2	-0.3<br>0.1	-0.1<br>0.4	-0.1<br>0.6	0.2<br>0.2	0.1<br>0.7	0.2<br>0.1	-0.46<br>0.002	0.1<br>0.5	0.0<br>0.9	-0.52<br>$7\cdot10^{-4}$	-0.1<br>0.6	0.1<br>0.5	-0.0<br>0.8	0.3<br>0.1
γH2AX	R1 %	-0.2<br>0.2	0.32<br>0.04	-0.0<br>0.8	0.0<br>0.8	0.2<br>0.2	-0.38<br>0.015		0.44<br>0.004	0.2<br>0.1	0.1<br>0.6	0.1<br>0.5	0.2<br>0.2	-0.2<br>0.2	-0.2<br>0.1	-0.2<br>0.2	-0.45<br>0.003	0.51<br>$9\cdot10^{-4}$	-0.3<br>0.1	-0.2<br>0.3	0.37<br>0.02	-0.0<br>0.9	-0.2<br>0.2	0.0<br>0.9	-0.46<br>0.003
	m	0.0<br>0.9	0.58<br>$1\cdot10^{-4}$	-0.1<br>0.6	0.1<br>0.4	0.2<br>0.3	-0.2<br>0.2	0.44<br>0.004		0.43<br>0.004	-0.1<br>0.5	0.2<br>0.2	0.2<br>0.1	0.3<br>0.1	0.0<br>0.9	0.1<br>0.5	-0.1<br>0.5	0.41<br>0.008	-0.44<br>0.005	0.0<br>0.8	0.54<br>$3\cdot10^{-4}$	0.0<br>0.9	0.0<br>1.0	-0.0<br>0.9	-0.2<br>0.3
	Index	-0.0<br>0.9	0.0<br>0.9	0.1<br>0.4	0.2<br>0.3	0.2<br>0.2	-0.0<br>0.9	0.2<br>0.1	0.43<br>0.004		-0.2<br>0.2	-0.0<br>0.9	0.1<br>0.7	0.2<br>0.1	-0.2<br>0.3	0.0<br>0.8	-0.1<br>0.5	0.1<br>0.4	-0.1<br>0.4	-0.1<br>0.6	-0.1<br>0.6	-0.1<br>0.8	0.1<br>0.7	0.43<br>0.005	-0.1<br>0.5
NRF2	RNA	0.78<br>$3\cdot10^{-9}$	-0.0<br>0.8	-0.0<br>0.7	0.0<br>1.0	-0.2<br>0.3	-0.2<br>0.2	0.1<br>0.6	-0.1<br>0.5	-0.2<br>0.2		0.0<br>0.9	0.0<br>0.9	0.0<br>0.9	0.0<br>0.9	-0.1<br>0.4	0.1<br>0.4	0.00<br>1.0	0.0<br>0.9	0.3<br>0.1	0.3<br>0.1	0.3<br>0.1	0.1<br>0.6	-0.1<br>0.4	0.1<br>0.5
	m	-0.0<br>0.7	0.64<br>$1\cdot10^{-5}$	0.1<br>0.4	0.0<br>0.9	0.1<br>0.7	-0.3<br>0.1	0.1<br>0.5	0.2<br>0.2	-0.0<br>0.9	0.0<br>0.9		0.55<br>$2\cdot10^{-4}$	0.0<br>0.9	-0.3<br>0.1	0.1<br>0.7	0.0<br>0.9	0.51<br>$9\cdot10^{-4}$	-0.2<br>0.1	-0.1<br>0.4	0.71<br>$2\cdot10^{-7}$	0.1<br>0.4	0.0<br>0.9	0.1<br>0.3	-0.1<br>0.5
	Index	-0.0<br>1.0	0.54<br>$3\cdot10^{-4}$	0.50<br>0.001	-0.1<br>0.6	0.1<br>0.5	-0.1<br>0.4	0.2<br>0.2	0.2<br>0.1	0.1<br>0.7	0.0<br>0.9	0.55<br>$2\cdot10^{-4}$		0.2<br>0.1	-0.2<br>0.2	-0.2<br>0.3	-0.0<br>0.9	0.36<br>0.02	-0.1<br>0.4	0.1<br>0.5	0.52<br>$7\cdot10^{-4}$	0.60<br>$4\cdot10^{-5}$	-0.1<br>0.4	0.2<br>0.2	-0.1<br>0.6
SOD1	RNA	0.47<br>0.002	0.0<br>0.9	-0.0<br>0.9	0.0<br>0.9	-0.40<br>0.011	-0.1<br>0.6	-0.2<br>0.2	0.3<br>0.1	0.2<br>0.1	0.0<br>0.9	0.0<br>0.9	0.2<br>0.1		0.0<br>0.8	-0.2<br>0.1	0.81<br>$1\cdot10^{-10}$	-0.2<br>0.2	0.0<br>0.9	0.44<br>0.005	0.0<br>0.8	0.2<br>0.2	0.3<br>0.1	0.3<br>0.1	0.2<br>0.4
	m	0.1<br>0.5	-0.1<br>0.6	-0.0<br>0.8	0.1<br>0.5	0.1<br>0.4	0.2<br>0.2	-0.2<br>0.1	0.0<br>0.9	-0.2<br>0.3	0.0<br>0.9	-0.3<br>0.1	-0.2<br>0.2	0.0<br>0.8		-0.2<br>0.1	0.1<br>0.6	0.1<br>0.7	0.2<br>0.2	0.2<br>0.4	-0.3<br>0.1	0.1<br>0.4	0.2<br>0.2	-0.41<br>0.009	-0.1<br>0.6
	Index	0.1<br>0.5	-0.0<br>0.7	0.1<br>0.4	0.1<br>0.5	0.1<br>0.5	0.1<br>0.7	-0.2<br>0.2	0.1<br>0.5	0.0<br>0.8	-0.1<br>0.4	0.1<br>0.7	-0.2<br>0.3	-0.2<br>0.1	-0.2<br>0.1		0.07<br>0.7	0.28<br>0.1	0.81<br>$1\cdot10^{-10}$	0.0<br>0.9	0.2<br>0.2	0.0<br>0.8	0.2<br>0.2	0.3<br>0.1	-0.3<br>0.1
HIF1A	RNA	0.51<br>$9\cdot10^{-4}$	0.42<br>0.006	0.3<br>0.1	0.1<br>0.4	-0.1<br>0.4	0.2<br>0.1	-0.45<br>0.003	-0.1<br>0.5	-0.1<br>0.5	0.1<br>0.4	0.0<br>0.9	-0.0<br>0.9	0.81<br>$1\cdot10^{-10}$	0.1<br>0.6	0.07<br>0.7		-0.36<br>0.02	0.1<br>0.4	0.62<br>$2\cdot10^{-5}$	-0.0<br>0.8	0.3<br>0.1	0.52<br>$7\cdot10^{-4}$	0.0<br>0.9	0.82<br>$1\cdot10^{-10}$
	m	-0.1<br>0.6	0.56<br>$2\cdot10^{-4}$	0.1<br>0.7	0.2<br>0.3	-0.46<br>0.002	-0.46<br>0.002	0.51<br>$9\cdot10^{-4}$	0.41<br>0.008	0.1<br>0.4	0.00<br>1.0	0.51<br>$9\cdot10^{-4}$	0.36<br>0.02	-0.2<br>0.2	0.1<br>0.7	0.28<br>0.1	-0.36<br>0.02		-0.1<br>0.6	-0.2<br>0.2	0.52<br>$7\cdot10^{-4}$	0.1<br>0.7	-0.33<br>0.03	-0.1<br>0.5	-0.2<br>0.3
	Index	-0.0<br>0.8	-0.0<br>0.8	-0.0<br>0.8	0.1<br>0.7	0.1<br>0.7	0.1<br>0.5	-0.3<br>0.1	-0.44<br>0.005	-0.1<br>0.4	0.0<br>0.9	-0.2<br>0.1	-0.1<br>0.4	0.0<br>0.9	0.2<br>0.2	0.81<br>$1\cdot10^{-10}$	0.1<br>0.4	-0.1<br>0.6		0.2<br>0.2	-0.2<br>0.2	0.2<br>0.1	0.3<br>0.1	0.0<br>0.9	0.2<br>0.2
BRCA1	RNA	0.60<br>$4\cdot10^{-5}$	0.0<br>0.9	-0.0<br>0.9	0.2<br>0.3	-0.1<br>0.6	0.0<br>0.9	-0.2<br>0.3	0.0<br>0.8	-0.1<br>0.6	0.3<br>0.1	-0.1<br>0.4	0.1<br>0.5	0.44<br>0.005	0.2<br>0.4	0.0<br>0.9	0.62<br>$2\cdot10^{-5}$	-0.2<br>0.2	0.2<br>0.2		-0.1<br>0.6	0.1<br>0.7	0.68<br>$3\cdot10^{-6}$	-0.1<br>0.4	0.81<br>$1\cdot10^{-10}$
	m	0.1<br>0.6	0.80<br>$1\cdot10^{-9}$	0.1<br>0.4	0.0<br>0.9	0.3<br>0.1	-0.52<br>$7\cdot10^{-4}$	0.37<br>0.02	0.54<br>$3\cdot10^{-4}$	-0.1<br>0.6	0.3<br>0.1	0.71<br>$2\cdot10^{-7}$	0.52<br>$7\cdot10^{-4}$	0.0<br>0.8	-0.3<br>0.1	0.2<br>0.2	-0.0<br>0.8	0.52<br>$7\cdot10^{-4}$	-0.2<br>0.2	-0.1<br>0.6		0.51<br>$9\cdot10^{-4}$	-0.0<br>0.9	-0.0<br>0.8	-0.1<br>0.5
	Index	0.54<br>$3\cdot10^{-4}$	0.42<br>0.006	0.50<br>0.001	-0.1<br>0.7	-0.1<br>0.7	-0.1<br>0.6	-0.0<br>0.9	0.0<br>0.9	-0.1<br>0.8	0.3<br>0.1	0.1<br>0.4	0.60<br>$4\cdot10^{-5}$	0.2<br>0.2	0.1<br>0.4	0.0<br>0.8	0.3<br>0.1	0.1<br>0.7	0.2<br>0.1	0.1<br>0.7	0.51<br>$9\cdot10^{-4}$		-0.1<br>0.4	0.1<br>0.6	0.38<br>0.01
BRCA2	RNA	0.64<br>$1\cdot10^{-5}$	0.1<br>0.5	0.3<br>0.1	0.1<br>0.1	-0.0<br>0.9	0.1<br>0.5	-0.2<br>0.2	0.0<br>1.0	0.1<br>0.7	0.1<br>0.6	0.0<br>0.9	-0.1<br>0.4	0.3<br>0.1	0.2<br>0.2	0.2<br>0.2	0.52<br>$7\cdot10^{-4}$	-0.33<br>0.03	0.3<br>0.1	0.68<br>$3\cdot10^{-6}$	-0.0<br>0.9	-0.1<br>0.4		-0.1<br>0.5	0.62<br>$2\cdot10^{-5}$
BAX	RNA	-0.0<br>0.8	-0.1<br>0.6	0.1<br>0.4	0.1<br>0.5	-0.42<br>0.007	-0.0<br>0.8	0.0<br>0.9	-0.0<br>0.9	0.43<br>0.005	-0.1<br>0.4	0.1<br>0.3	0.2<br>0.2	0.3<br>0.1	-0.41<br>0.009	0.3<br>0.1	0.0<br>0.9	-0.1<br>0.5	0.0<br>0.9	-0.1<br>0.4	-0.0<br>0.8	0.1<br>0.6	-0.1<br>0.5		-0.1<br>0.5
BCL2	RNA	0.71<br>$2\cdot10^{-7}$	0.0<br>0.9	-0.0<br>0.8	0.2<br>0.2	0.1<br>0.6	0.3<br>0.1	-0.46<br>0.003	-0.2<br>0.3	-0.1<br>0.5	0.1<br>0.5	-0.1<br>0.5	-0.1<br>0.6	0.2<br>0.4	-0.1<br>0.6	-0.3<br>0.1	0.82<br>$1\cdot10^{-10}$	-0.2<br>0.3	0.2<br>0.2	0.81<br>$1\cdot10^{-10}$	-0.1<br>0.5	0.38<br>0.01	0.62<br>$2\cdot10^{-5}$	-0.1<br>0.5	
